# Correction: Epidemiological and health economic implications of symptom propagation in respiratory pathogens: A mathematical modelling investigation

**DOI:** 10.1371/journal.pcbi.1012791

**Published:** 2025-01-29

**Authors:** Phoebe Asplin, Matt J. Keeling, Rebecca Mancy, Edward M. Hill

In Fig 5, the stacked bar heights normalization is incorrect. The height of each stacked bar should be 1.0 instead of 0.8. Additionally, there is an error in the legend. The shade red should be associated with "Recovered Severe Infected Case (R_S_)" and yellow with "Recovered Mild Infected Case (R_M_)". Please see the correct Fig 5 and captions here.

**Fig 5:**
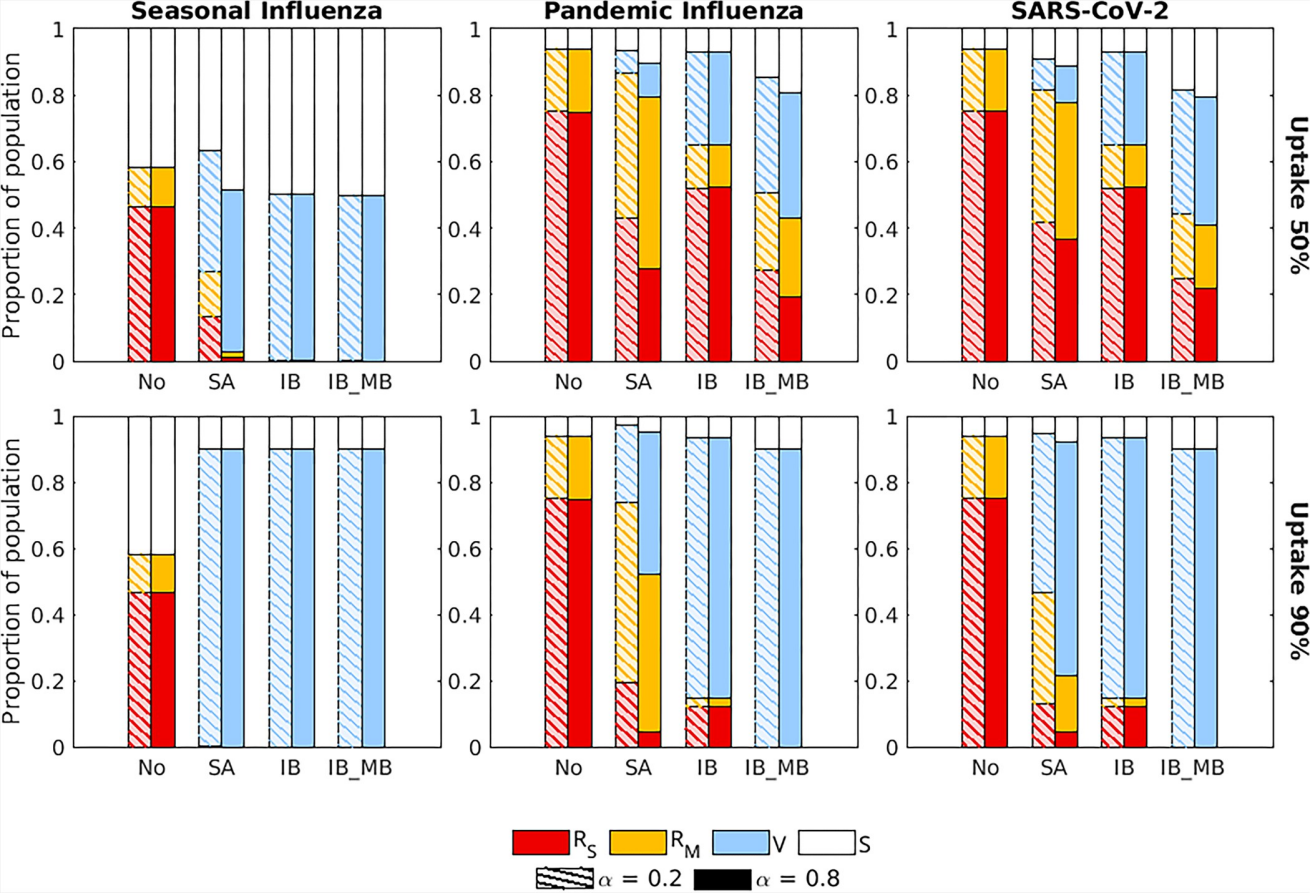
The proportion of the population in each disease state at the end of the outbreak for the four intervention scenarios, two vaccine uptake levels and three disease parameterisations. The four groups of bars correspond to four intervention scenarios: no intervention (No), a symptom-attenuating vaccine (SA), an infection-blocking vaccine (IB) and an infection-blocking vaccine which only admits mild breakthrough infections (IB_MB). The two bars in each group correspond to two different strengths of symptom propagation: *α* = 0.2 (left bar with hatched lines) and *α* = 0.8 (right bar with solid fill). Bar shading corresponds to the disease status: red—recovered from severe infection (*R*_*S*_); yellow—recovered from mild infection (*R*_*M*_); blue—susceptible and vaccinated (*V*); white—susceptible and not vaccinated (*S*). The two rows correspond to two vaccine uptake levels: **(A-C)** 50%; **(D-F)** 90%. Columns correspond to different disease parameterisations: **(A,D)** seasonal influenza; **(B,E)** pandemic influenza; **(C,F)** SARS-CoV-2. We fixed the vaccine efficacy at 70% and all other parameters as given in Table 1, with *ν* chosen to fix the proportion of cases that were severe equal to 0.8.

In S1 Text, there are errors in the legend of the Figures D, L, P, and T. The shade red should correspond to "Recovered severe infected case (R_S_)" and yellow to "Recovered mild infected case (R_M_)". Please view the correct S1 Text below.

## Supporting information

S1 Text Supporting information for ‘Epidemiological and health economic implications of symptom propagation in respiratory pathogens: A mathematical modelling investigation’. This supplement consists of the following parts: (1) Methods for calculating *R*_0_ and *β*; (2) Results with fixed *β*; (3) Parameterisation details of the health economic model; (4) Results for an alternative vaccine action; (5) Additional epidemiological results; (6) Sensitivity to discounting; (7) Sensitivity to intervention efficacy; (8) Additional health economic findings.
(PDF)
