# Supplementary material for: Epidemiological and health economic implications of symptom propagation in respiratory pathogens: A mathematical modelling investigation
Source: PLoS Comput Biol. 2024 May 3;20(5):e1012096. doi: 10.1371/journal.pcbi.1012096 (PMC11095726; doi:10.1371/journal.pcbi.1012096)
Supplement: Attachment — Submitted filename: Response to Reviewers_ Symptom propagation modelling paper.pdf [file pcbi.1012096.s002.pdf]

# Response to Reviewers

**Epidemiological and health economic implications of symptom propagation in respiratory pathogens: A mathematical modelling investigation** by P. Asplin, R. Mancy, M. J. Keeling & E. M. Hill.

*Herein we provide a detailed response to each of the Reviewers' comments in blue italics interspersed with the original comments in black font.*
**Where appropriate, at the end of a comment we state in bold red font and within parentheses the line numbers where that edit/addition can be found within the revised manuscript (clean copy and tracked changes copy)**

## Editorial assessment

As with all papers reviewed by the journal, your manuscript was reviewed by members of the editorial board and by several independent reviewers. In light of the reviews (below this email), we would like to invite the resubmission of a significantly-revised version that takes into account the reviewers' comments.

Reviewers have raised quite some important questions about how (biologically and clinically) realistic some parameters in the model are. Authors should seriously consider these and address reviewers' concerns as provided below.

***Authors' response:***
*Thank you for the careful consideration of our manuscript. We are appreciative of the comments to steer our revisions regarding revisions to improve our article. Through our responses to the individual comments raised by the reviewers (detailed below), we believe we have addressed these items in our revised submission.*

## Reviewer 1

In this paper, the authors proposed to introduce a parameter alpha to model the symptom propagation of respiratory infections in a modified SEIR model and applied this model to three scenarios of respiratory infectious diseases, including seasonal influenza, pandemic influenza, and SARS-CoV-2. While the manuscript is well written and the methods are clearly documented, it is not clear to me how the proposed integration of the alpha parameter made a new contribution to the existing modelling framework. Please see below for my specific comments.
***Authors' response:***
*We thank the reviewer for the positive summary of the presentation of our manuscript. We appreciate them sharing their perspective, which through our subsequent edits our intent has been to enhance the novelty and implications of the results.*

1. It is understandable to model the symptom propagation by a single parameter alpha ranging from 0 to 1. However, it is not clear what this alpha means clinically and how it could be applied to the design of control strategies. For example, given that there was ample data from the COVID-19 pandemic, what is the estimated alpha for Omicron BA.1 in early 2022 in

the UK? If the alpha parameter were to be estimated for the Omicron outbreak, how would the control strategies be optimized?

***Authors' response:***

*Estimation of the alpha parameter is non-trivial and requires a significant amount of individual-level data on who infected whom and the severity of their symptoms. This process is further complicated by the existence of asymptomatic infections that may be undetected within the system. We are in close discussions with data holders and hope to undertake this parameter inference work soon.*

*Once alpha is determined, it can inform the relative importance of transmission from those who are asymptomatic and therefore the optimal approach for contact tracing. It will also help inform the role of vaccines, which may reduce symptom severity in addition to reducing the level of infection.*

*We agree with the reviewer that this is an important question to consider, and hence have expanded upon our discussion to highlight that the inference of the alpha parameter is a key area of future work.*

"Another identified key area of future work is the estimation of the alpha parameter, i.e. the strength of symptom propagation. However, due to the complex nature of symptom severity and the many confounding factors, performing this inference is non-trivial. A large volume of individual data with both information on symptom severity and who infected whom is required. Major challenges include separating symptom propagation from the effects of strains and from the impact of genetic similarity between an individual and the person who infected them (e.g. in the case of related individuals). Nevertheless, the public health benefits of such estimations will make surmounting such challenges rewarding, including informing the relative importance of transmission from those who are asymptomatic (and therefore the optimal approach for contact tracing) and the role of vaccines that may reduce symptom severity as well as infection burden. Close dialogue with appropriate data holders will be a crucial aspect to successfully accomplish these goals."

**(Clean copy: lines 620-630)**

**(Tracked changes: lines 626-636)**

2. Many of the results are expected. For example, on Page 6, the two beta (i.e., beta_M and beta_S) were calibrated such that the stated value of the basic reproductive number R_0 was acquired. Therefore, it is not surprising that the final cumulative infection attack rates were similar across different values of the alpha parameter. Thus, the strategies reducing symptoms or severity would be more effective with the increasing value of alpha.

***Authors' response:***

*To some degree, we agree with this statement. It is intuitive that strategies reducing symptoms or severity would be more effective under increasing strengths of symptom propagation. However, we believe that the demonstration of these intuitive results through mathematical modelling is an important step in strengthening the knowledge base surrounding symptom propagation. This is especially pertinent given that models including symptom propagation are understudied, and it is almost never considered when evaluating intervention strategies.*

*In regard to the specific example given, we think that it is important to adopt a modelling strategy that aligns with approaches to real-world infections. In such practical contexts, R_0 is the parameter most likely to be known; other model parameters (in this case beta) would be inferred to generate the measured R_0 value, and then the impact of alpha*

*(and any further parameters) assessed. This is the approach we have adopted here. We now state in the Methods:*

*"To better align with approaches taken when analysing real-world infections, we chose to fix R_0, as opposed to fixing the value of beta; R_0 is the parameter most likely to be known from available empirical measurements, with other model parameters (in this case beta) inferred to generate the measured R_0 value."*

**(Clean copy: lines 168-171)**
**(Tracked changes: lines 168-172)**

*We agree that it is not necessarily surprising that the final cumulative infection attack rates were similar across different values of the alpha parameter. However, it is not uncommon for the final cumulative infection attack rates to vary with certain parameters despite R_0 being fixed when model extensions are applied. One notable example is in network models, in which the size of an outbreak can vary appreciably with network structure under fixed R_0* (Hébert-Dufresne, L., Althouse, B.M., Scarpino, S.V. and Allard, A., 2020. Beyond R 0: heterogeneity in secondary infections and probabilistic epidemic forecasting. Journal of the Royal Society Interface, 17(172), p.20200393).

*In addition, we perceive the similar final cumulative infection attack rates as a benefit of fixing the value of R_0. By taking this approach we have been able to separate the effects of symptom propagation from the effects of an increase in total cases. If we had chosen to fix beta instead of R_0, then the final cumulative infection attack rate would have increased strongly with alpha and all strategies would have a greater effect for stronger symptom propagation.*

*To demonstrate this, we have added further supplementary figures showing our results with fixed beta instead of fixed R_0. In these scenarios, the value of beta was chosen for each disease parameterisation (seasonal flu, pandemic flu, SARS-CoV-2) to give the same value of R_0 as in the main text under the condition where alpha = 0. We find that R_0 dramatically increases with alpha (Fig S1). This is particularly clear for SARS-CoV-2, for which R_0 = 10 when alpha = 1 (Fig S1 (c)). Thus, our model would no longer be appropriate for our chosen disease parameterisations at these higher values of alpha, because we know that these large values of R_0 are unrealistic. In addition, our new analysis shows that under fixed beta, increasing alpha results in a notable increase in both the final outbreak size and the peak prevalence (Fig S2). For example, we found that for seasonal influenza, the final outbreak size ranged from close to 60% of the population being infected for alpha = 0, to over 90% for alpha = 1 (Fig S2 (a)). Due to this increase in both R_0 and the final outbreak size for fixed beta, we would expect all interventions to be more effective at reducing the number of cases for larger values of alpha.*

**(Fixed beta supplementary plots: Figs S1 & S2)**

3. Throughout the paper, the symptom propagation was modeled in a mechanism similar to the "all-or-nothing" mechanism in vaccines. For example, on Page 9, it was assumed that vaccinated individuals have probability eta of having mild disease and probability (1-eta) to be determined as usual severity. How would the model be modified if the symptom propagation functions in a way similar to the "leaky" mechanism in vaccines?

***Authors' response:***

*In our view, an "all-or-nothing" vaccine would be one for which a subset of vaccinated individuals have full protection and the remainder have none. In contrast, a "leaky" vaccine would be one in which each vaccinated individual has a "level" of protection (between 0% and 100%) such that it would prevent infection in a corresponding proportion of exposure events. Another way of considering the differences is that for the all-or-nothing vaccine, its continuing impact is set at the time of vaccination, whereas for the leaky vaccine, its impact is randomly assigned each time there is a challenge. The distinction between all-or-nothing and leaky vaccine actions is therefore only important when there exist multiple infectious challenges (or more generally there are multiple time points at which a stochastic transition can occur).*

*Analogously, for the symptom propagation mechanism, the distinction between an all-or-nothing and leaky action would only arise when repeat infections are possible (multiple time points at which a stochastic transition for determination of symptom severity can occur). However, under our modelling assumptions, it is not possible for members of the population to contract infection more than once. Therefore, the distinction between a leaky or all-or-nothing symptom propagation mechanism is not relevant under such an infection assumption.*

*Though interesting, presently we have not added these details to the paper. We feel a full description of these technicalities in the paper would disrupt the flow of the text, detracting from the main narrative of the study.*

What if there is a severity spectrum and there is not a clear cut between "mild", "usual" and "severe"?

***Authors' response:***

*We agree that severity is a spectrum and future work aims to more fully recognise this concept. Our model can be extended to a larger number of severity categorisations, with our single value of alpha being replaced with a matrix of probabilities of having severity level i, given being infected by someone with severity level j. The framework may be further generalised to approximate a continuous severity spectrum by the use of a stochastic model with a severity probability distribution that shifts according to the severity level of the infector. An alternative approach to represent a continuous severity spectrum would be formulating the model as a set of PDEs, but that has associated numerical and computational overheads.*

*Whilst these are interesting extensions to our model, both of these models would be much more complex and would make it more difficult to encapsulate the strength of symptom propagation in a single parameter. Having fewer model parameters has benefits for the clarity of findings and will be more amenable for parameter estimation. We have now elaborated in the Discussion on how potential model extensions can include non-trivial mappings from symptom sets to severity and how, ultimately, informing an appropriate model structure should be guided by data:*

*"An extension of this model to include a separate asymptomatic class or a continuum of severity could be explored in future work (or even qualitatively different symptom sets that do not straightforwardly map to levels of severity), with model structural choices informed by data where appropriate."*

**(Clean copy: lines 592-595)**

**(Tracked changes: lines 598-601)**

## Reviewer 2

Asplin et al. developed a mathematical modelling framework incorporating symptom propagation and applying to a range of pathogens for investigating the epidemiological and health-economic implications of symptom propagation. The research design is appropriate and the results and methods are clearly described.

***Authors' response:***

*We thank the reviewer for the appraisal of our study and the kind words on its clarity.*

I have some further suggestions that the authors could improve or discuss. Specially,

1. (Major) The authors should provide a more detailed presentation of their data source, at least, the year of data collection, the country from which the data originates, and the particular viral subtypes/clades/lineages involved. Those information can play a pivotal role in the interpretation and validity of the model's outcomes. Is there a possibility to incorporate spatiotemporal data into this study? It might provide further insights or depth to the findings.

***Authors' response:***

*The intent of our study is to demonstrate a novel mathematical framework, and that symptom propagation has the potential to notably impact epidemiological outcomes and the cost-effectiveness of intervention strategies. In this paper, we have used published estimates for the epidemiological parameters to calibrate the model. We view fitting the model to a dataset as a key area of future work, both for more accurately estimating the general model parameters and for inferring alpha to determine the extent to which symptom propagation occurs. We have added an additional paragraph to the discussion in which this point is highlighted (see also our response to Comment 1 from Reviewer 1).*

**(Clean copy: lines 620-630)**

**(Tracked changes: lines 626-636)**

*As we have not used a dataset to fit our model to data, providing the level of detail described by the reviewer for each parameter and literature source is beyond the scope of the manuscript; full citations are provided in Table 1, and the reader can refer back to the original sources for this information. However, we acknowledge that these parameters can vary widely between subtypes or strains of viruses. Hence, we have clarified in our methods that the parameters for influenza are based on studies of influenza A, and the parameters for SARS-CoV-2 are based on early estimates for the wild-type virus.*

*"These values of R_0 were chosen to reflect estimates in the literature of 1-1.69 for seasonal influenza, 1.95-3.5 for pandemic influenza and 2.43-3.60 for wild-type SARS-CoV-2 … Estimates for the other parameters were taken from studies of influenza A virus strains and estimates for wild-type SARS-CoV-2 … (full details in Table 1)."*

**(Clean copy: lines 164-178)**

**(Tracked changes: lines 164-179)**

2. (Major) Related approaches should be incorporated into this study and applied to the same dataset for the comparison.

***Authors' response:***

*Our view is that the main related approach would be the same model with no symptom propagation, which is the standard benchmark model in the epidemiological literature. This is equivalent to our model with alpha=0. In our discussion, we mention further extensions to the model framework that would warrant their own dedicated study, including stochastic models and network or spatial models. However, these extensions are outside of the scope of the present study which aims to explore the effect of symptom propagation in a relatively simple, well-understood model.*

3. Supporting Information from S12 to S21 are absent from the main text.

***Authors' response:***

*We thank the reviewer for highlighting the omission of references to these supporting information figures in the main text. We have updated the main text to reference these figures.*

**(Clean copy: lines 291, 335, 379)**
**(Tracked changes: lines 296, 341, 385)**

4. For the reproducibility, it would be helpful to have clear instructions for running the code provided in their Github repository.

***Authors' response:***

*We have improved the clarity of the instructions in the README file in the Github repository and added additional comments within the files to specify what the run sets are for and which figures are created from the output generated.*

5. Please indexing all the equations throughout the paper to help readers quickly locate specific equations.

***Authors' response:***

*We thank the reviewer for their suggestion and have indexed all of the equations.*

## Reviewer 3

The authors proposed a framework to incorporate symptom propagation in the transmission of seasonal, pandemic flu and COVID. The framework was nicely developed and presented. My main comments are about the consistency of parameters for SARS-CoV-2 variant and interpretation of the results.

***Authors' response:***

*We thank the reviewer for the thoughtful comments provided on our manuscript. Through our amendments in response to each of the comments below, we have endeavoured to bolster our justification of the SARS-CoV-2 parameters and enhance our acknowledgement of how our findings may be impacted by our modelling assumptions.*

Major comments:

1. Method, would the authors comment on whether the analyses should fix R0, or fix beta and let R0 to vary to draw the most meaningful conclusion?

***Authors' response:***

*We believe that it is important to adopt a modelling strategy that aligns with approaches to studying real-world infections. In such practical applications, R_0 is the parameter most likely to be known, with other model parameters (in this case beta)*

*calibrated to generate the observed R_0 value. The impact of the variables of interest on the infection transmission dynamics (such as alpha in our case) can then be assessed. This is the approach we have adopted here.*

*To allow for comparison between the two methods we have added an additional Supporting Information section that demonstrates the impact of alpha on epidemiological outcomes under fixed beta. We found that the value of R_0 and the final outbreak size varied appreciably with alpha (Figs S1 & S2, see also our response to Comment 2 from Reviewer 1). The effect of total cases increasing with alpha makes it difficult to compare other outcomes such as the proportion of cases that are severe. In particular, it would be difficult to explore the impact of the strength of symptom propagation on intervention effectiveness, as more cases would always be prevented for higher alpha values.*

**(Supporting Information: Section S2)**

2. There are significant differences in the epidemiological and clinical parameters between pre-omicron and omicron SARS-CoV-2 variants. Would the authors consider harmonizing all SARS-CoV-2 parameters in Table 2 and 3?

***Authors' response:***

*We thank the reviewer for their suggestion. Due to the substantial heterogeneity between variants, we agree that this is an important clarification to make. We have now explicitly stated that all parameters are for wild-type SARS-CoV-2.*

*"Estimates for the other parameters were taken from studies of influenza A virus strains and estimates for wild-type SARS-CoV-2."*

**(Clean copy: lines 173-174)**

**(Tracked changes: lines 174-175)**

3. R0 = 3 for SARS-CoV-2 tended to be low even for pre-omicron variants

***Authors' response:***

*Whilst we agree that this estimate would be low for omicron and potentially other variants, we think that this is a reasonable estimate for the first wave of wild-type infections. Indeed, for the early stages of the epidemic in Italy, R_0 was estimated to be between 2.43-3.10 (*D'Arienzo, M. and Coniglio, A., 2020. Assessment of the SARS-CoV-2 basic reproduction number, R0, based on the early phase of COVID-19 outbreak in Italy. Biosafety and Health, 2(2), pp.57-59.*), and a review of early estimates of R_0 in China found an average value of 3.28 and a median value of 2.79 (*Liu, Y., Gayle, A.A., Wilder-Smith, A. and Rocklöv, J., 2020. The reproductive number of COVID-19 is higher compared to SARS coronavirus. Journal of travel medicine.*).*

*We have updated the text to specify that we are considering wild-type SARS-CoV-2 and to include references for estimates of R_0 in the literature.*

*"These values of R_0 were chosen to reflect estimates in the literature of 1-1.69 for seasonal influenza, 1.95-3.5 for pandemic influenza and 2.43-3.60 for wild-type SARS-CoV-2 "*

**(Clean copy: lines 164-166)**

**(Tracked changes: lines 164-166)**

4. Severe patients are more likely to be immobile and reduce contacts with others, while very mild patients are more likely to maintain daily activities. Were the transmission

parameters in Table 2 (2x and 4x transmissibility for flu and SARS-CoV-2) assumed they have same or different contact pattern?

***Authors' response:***

*Our parameters were chosen assuming that mild and severe patients have the same contact patterns. We agree with the reviewer that this is an important consideration and have expanded our discussion of the limitations of our work to highlight this point.*

*"A notable limitation of these choices is that the transmission rates do not account for changes in contact patterns that we would expect to see for those with more severe disease, and hence may be an overestimation of the real values. In future work, it would be valuable to include heterogeneity in contact patterns as a function of symptom severity."*

**(Clean copy: lines 570-57)**

**(Tracked changes: lines 576-581)**

5. Discussion, the analysis has fixed R0 and adjust beta to keep R0 constant. However, in practice the change in symptom propagation parameters may modify R0 via disease severity and corresponding contact pattern.

***Authors' response:***

*We agree with the reviewer that the change in symptom propagation parameters would modify the value of R_0. We have updated the text to specify that for each value of alpha we computed the value of beta to obtain the specified R_0 value for that pathogen:*

*"To obtain these fixed values of R_0, we computed the required value of the transmission rate beta for each value of alpha by deriving an equation for R_0 using the next-generation matrix approach"*

**(Clean copy: lines 166-168)**

**(Tracked changes: lines 166-168)**

*We have also added additional supplementary figures showing how the value of R_0, final outbreak size and peak prevalence vary with alpha, assuming a fixed value of beta (see also our response to Comment 2 from Reviewer 1).*

**(Supporting Information: Section S2)**
