# Supplementary material for: Correction: Epidemiological and health economic implications of symptom propagation in respiratory pathogens: A mathematical modelling investigation
Source: PLoS Comput Biol. 2025 Jan 29;21(1):e1012791. doi: 10.1371/journal.pcbi.1012791 (PMC11778760; doi:10.1371/journal.pcbi.1012791)
Supplement: S1 Text — This supplement consists of the following parts: (1) Methods for calculating R0 and β; (2) Results with fixed β; (3) Parameterisation details of the health economic model; (4) Results for an alternative vaccine action; (5) Additional epidemiological results; (6) Sensitivity to discounting; (7) Sensitivity to intervention efficacy; (8) Additional health economic findings. (PDF) [file pcbi.1012791.s001.pdf]

S1 Text
Epidemiological and health economic implications of symptom propagation in respiratory pathogens: A mathematical modelling investigation

Phoebe Asplin[1,2,3*], Matt J. Keeling[2,3,4], Rebecca Mancy[5,6], Edward M. Hill[2,3*]

1 EPSRC & MRC Centre for Doctoral Training in Mathematics for Real-World Systems, University of Warwick, Coventry, United Kingdom.
2 Mathematics Institute, University of Warwick, Coventry, United Kingdom.
3 The Zeeman Institute for Systems Biology & Infectious Disease Epidemiology Research, University of Warwick, Coventry, United Kingdom.
4 School of Life Sciences, University of Warwick, Coventry, United Kingdom.
5 School of Biodiversity, One Health and Veterinary Medicine, University of Glasgow, University Avenue, Glasgow, United Kingdom.
6 MRC/CSO Social and Public Health Sciences Unit, University of Glasgow, Clarice Pears Building, 90 Byres Road, Glasgow, United Kingdom.

* Corresponding Authors. Emails: p.asplin@warwick.ac.uk; Edward.Hill@warwick.ac.uk.

Table of Contents

1 Calculating the basic reproduction number, $\mathbf{R_0}$, and calibrating β 2

2 Results with fixed β 3

3 Health economic model: Parameterisation details 5
3.1 Likelihood of hospitalisation and death 5
3.2 Quality-adjusted life years (QALYs) 5
3.3 Monetary costs 5
3.4 Discounting 5

4 Alternative infection-blocking and modified breakthrough infection intervention 6
4.1 Modified model equations 6
4.2 Simulation overview 7
4.3 Summary of findings 7

5 Additional epidemiological outcomes 10
5.1 Final outbreak size and peak prevalence 10
5.2 Temporal intervention plots 11
5.3 Additional intervention bar plots 14
5.4 Outcomes conditional on $\boldsymbol{\nu} = 0.2$ 16

6 Sensitivity to discounting 18

7 Sensitivity to intervention efficacy 21
7.1 50% efficacy 22
7.2 90% efficacy 26

8 Additional health economic findings **30**
8.1 Relative threshold unit intervention cost 30
8.2 Most cost-effective intervention uptake 33
8.3 Implication on threshold unit intervention cost when accounting for symptom propagation 36

1 Calculating the basic reproduction number, $\mathbf{R_0}$, and calibrating $\boldsymbol{\beta}$

The basic reproductive number, R_0, is commonly used in epidemiology to indicate a disease's potential to spread through a population. It is defined as the average number of secondary cases generated by an average infectious individual in a fully susceptible population. R_0 can be formulated as

$$R_0 = (\text{average number of infections generated per unit time}) \times (\text{average infectious period}).$$

We calculated the value of R_0 using the next-generation matrix (NGM) approach. This method was developed by Diekmann *et al.* [1] to calculate the value of R_0 in heterogeneous populations where compartments are split into a finer structure (e.g. into age classes). The next generation matrix is defined to be

$$\mathbf{K} = \begin{pmatrix} k_{11} & \cdots & k_{1n} \\ \vdots & & \vdots \\ k_{n1} & \cdots & k_{nn} \end{pmatrix}$$

where k_{ij} is the average number of type-i cases generated by a type-j case in a fully susceptible population. This can be applied to the ODE model by considering a vector containing the number of people in each infectious class, which then multiplies by $\mathbf{K}$ at each time step. This vector grows at a rate given by the dominant eigenvalue of $\mathbf{K}$ - the eigenvalue with the largest absolute value - with R_0 being this dominant eigenvalue.

In the case of our model,

$$\mathbf{K} = \begin{pmatrix} k_{MM} & k_{MS} \\ k_{SM} & k_{SS} \end{pmatrix} = \begin{pmatrix} \frac{\beta_M}{\gamma_M}(\alpha + (1-\alpha)(1-\nu)) & \frac{\beta_S}{\gamma_S}(1-\alpha)(1-\nu) \\ \frac{\beta_M}{\gamma_M}(1-\alpha)\nu & \frac{\beta_S}{\gamma_S}(\alpha + (1-\alpha)\nu) \end{pmatrix}.$$

R_0 is an eigenvalue of $\mathbf{K}$ and thus solves

$$\begin{aligned} 0 &= \lambda^2 - (k_{MM} + k_{SS})\lambda + (k_{MM}k_{SS} - k_{MS}k_{SM}) \\ &= \lambda^2 - \left(\frac{\beta_M}{\gamma_M}(\alpha + (1-\alpha)(1-\nu)) + \frac{\beta_S}{\gamma_S}(\alpha + (1-\alpha)\nu) \right) \lambda + \frac{\beta_M \beta_S}{\gamma_M \gamma_S}\alpha. \end{aligned}$$

If $\beta_M = \beta$, $\beta_S = r\beta$, then the derived equation for R_0 simplifies to

$$\frac{r\alpha}{\gamma_M \gamma_S}\beta^2 - R_0 \left(\frac{(1-(1-\alpha)\nu)}{\gamma_M} + \frac{r(\alpha + \nu - \alpha\nu)}{\gamma_S} \right) \beta - R_0^2 = 0,$$

from which, for a given value of R_0, we calculated the required value of β.

2 Results with fixed β

In the main text we fixed the value of R_0 for each parameter set by choosing an appropriate value of β for each value of α. Here we show our results with fixed β instead. For each of the three disease parameterisations we chose β_M to give the stated value of R_0 when $\alpha = 0$: $R_0 = 1.5$ for seasonal influenza, $R_0 = 3.0$ for pandemic influenza and SARS-CoV-2.

We found that the value of R_0 increased with α (Fig. A) and similarly with the total outbreak size and peak prevalence (Fig. B). As with our main text results, the proportion of cases that were severe increased with α (Fig. B). However, the outbreak duration decreased with α (Fig. B)

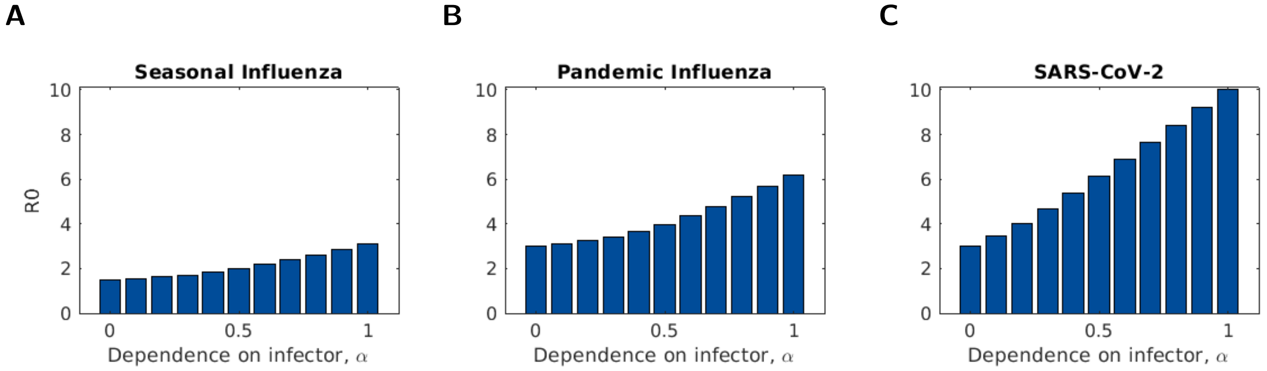

Figure A: The basic reproduction number, R_0, for three disease parameterisations, plotted for different symptom propagation strengths, α and fixed values of β. In all panels, $\nu = 0.2$. The three disease parameterisations used were: **(a)** Seasonal influenza; **(b)** Pandemic influenza; **(c)** SARS-CoV-2, with parameters as given in Table 1. For each of the three disease parameterisations we chose β_M to give the stated value of R_0 when $\alpha = 0$: $R_0 = 1.5$ for seasonal influenza, $R_0 = 3.0$ for pandemic influenza and SARS-CoV-2.

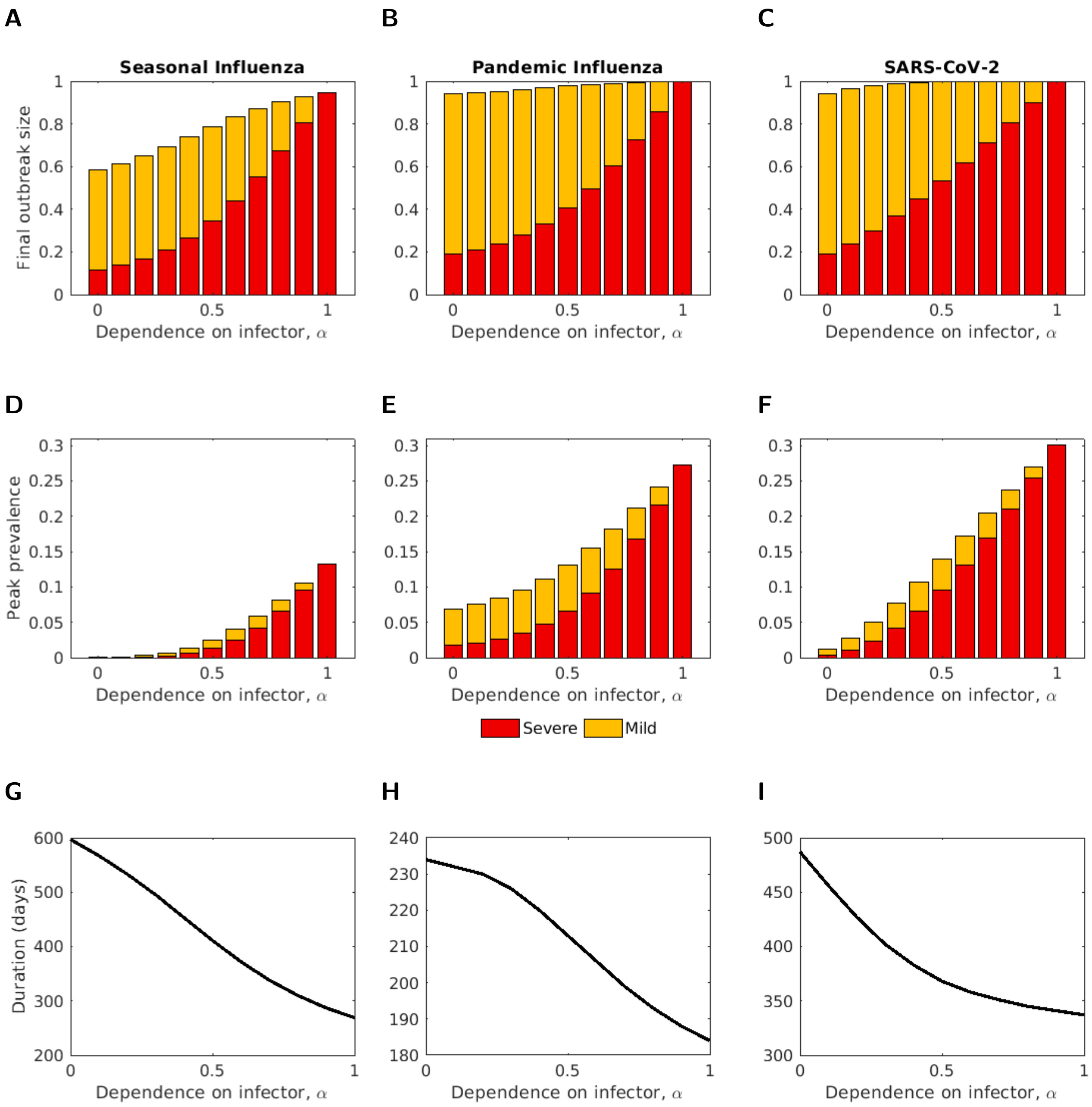

Figure B: The final outbreak size, peak prevalence and outbreak duration, by severity, for three disease parameterisations, plotted for different symptom propagation strengths, α and fixed values of β. (a-c) Final outbreak size by severity. **(d-f)** Peak prevalence by severity. The intensity of the shading denotes the symptom severity class, with severe cases in red and mild cases in yellow. **(g-i)** Outbreak duration (note the different y-axis scales). In all panels, $\nu = 0.2$. The three disease parameterisations used were: **(a,d,g)** Seasonal influenza; **(b,e,h)** Pandemic influenza; **(c,f,i)** SARS-CoV-2, with parameters as given in Table 1. For each of the three disease parameterisations we chose β_M to give the stated value of R_0 when $\alpha = 0$: $R_0 = 1.5$ for seasonal influenza, $R_0 = 3.0$ for pandemic influenza and SARS-CoV-2.

3 Health economic model: Parameterisation details

3.1 Likelihood of hospitalisation and death

Hospitalisation and death rates were taken from Hill *et al.* [2] for influenza and Moran *et al.* [3] for SARS-CoV-2.

3.2 Quality-adjusted life years (QALYs)

We assumed that mild disease had sufficiently minimal symptoms, thereby causing no QALY losses.

We took influenza QALY losses for severe, non-fatal cases were taken from Hill *et al.* [2]. For SARS-CoV-2, we used estimates for years lived with disability (YLD) per case from Moran *et al.* [3]. We used the YLD for "severe" (non-ICU) and "critical" (ICU) hospitalised cases to estimate QALY losses for non-fatal hospitalised cases. We used the YLD for "moderate" and "post-acute consequences" to estimate QALY losses for severe non-hospitalised cases.

The QALY losses per death depend primarily on the number of healthy years lost and therefore the age of the individual. Our model was not stratified by age class, so to estimate the average QALY losses per death, we used estimates of the total QALY losses from deaths during an outbreak and divided by the number of deaths. For influenza, we used data from an outbreak in Spain during the 2009 H1N1 pandemic [4]. For SARS-CoV-2, we used a study of COVID-19 cases in the Republic of Ireland from March 2020 to February 2021 [3].

3.3 Monetary costs

Hospital costs for influenza were taken from Hill *et al.* [2]. Hospital costs for SARS-CoV-2 were estimated under the assumption that costs would be twice as much as influenza, due to the average length of stay being roughly twice as long for SARS-CoV-2 (around 13 days [5]) than for influenza (around 7 days [6]).

3.4 Discounting

A common component of health economic modelling is discounting, which assigns a lower value to costs and health outcomes that occur in the future [7]. For the scenarios where we applied a discounting rate of 3.5%, the discounted value (of QALYs lost or monetary costs, as appropriate) in year y was given by

$$\text{Discounted value}(y) = \text{Raw value}(y) \left(\frac{1}{1 + 0.035} \right)^{y-1}.$$

4 Alternative infection-blocking and modified breakthrough infection intervention

Throughout this section, we modelled an infection-blocking vaccine for which breakthrough infections were only possible when the infector was a severe case (Fig. C).

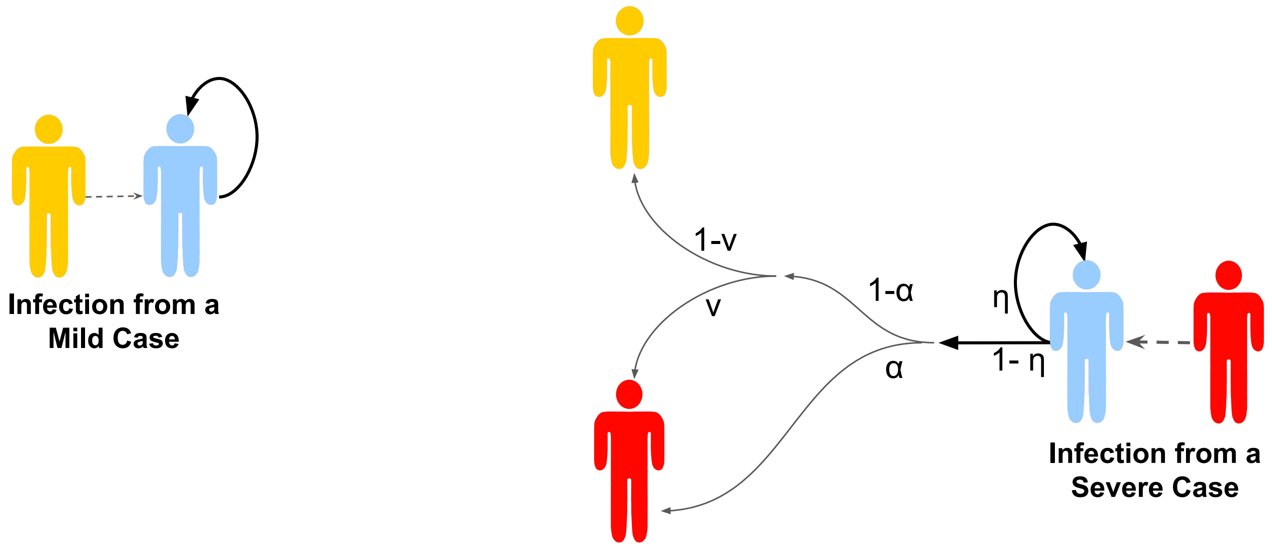

Figure C: Schematic of the infection-blocking and modified breakthrough infection vaccine. If challenged by infection from a mild case, a vaccinated individual would be guaranteed to be protected. If challenged by infection from a severe case, a vaccinated individual had a probability η of their infection being prevented and a probability $1 - \eta$ of being infected, with their symptom severity then being determined as usual. Yellow shaded individuals correspond to infectious cases with mild symptoms, red shaded individuals correspond to infectious cases with severe symptoms and blue shaded individuals correspond to those who are vaccinated. The values on the arrows show the corresponding probability.

4.1 Modified model equations

Under the use of such an intervention, the model dynamics are governed by the following system of ODEs. Recall that the V class denotes those who were susceptible and vaccinated, whilst we denote with red font those terms that include the action of the intervention:

$$
\begin{aligned}
\frac{dS}{dt} &= -(\lambda_M + \lambda_S)S \\
\frac{dV}{dt} &= -(1-\eta)\lambda_S V \\
\frac{dE_M}{dt} &= (\alpha + (1-\alpha)(1-\nu))\lambda_M S + (1-\alpha)(1-\nu)\lambda_S\Big(S + (1-\eta)V\Big) - \epsilon E_M \\
\frac{dE_S}{dt} &= (1-\alpha)\nu\lambda_M S + (\alpha + (1-\alpha)\nu)\lambda_S\Big(S + (1-\eta)V\Big) - \epsilon E_S \\
\frac{dI_M}{dt} &= \epsilon E_M - \gamma_M I_M \\
\frac{dI_S}{dt} &= \epsilon E_S - \gamma_S I_S \\
\frac{dR_M}{dt} &= \gamma_M I_M \\
\frac{dR_S}{dt} &= \gamma_S I_S
\end{aligned}
$$

where the force of infection from mild cases, λ_M, and severe cases, λ_S, respectively, were given by:

$$
\lambda_M = \frac{\beta_M I_M}{N}, \quad \lambda_S = \frac{\beta_S I_S}{N}.
$$

4.2 Simulation overview

As for the interventions studied in the main text, we ran a range of values of α and ν (both between 0 and 1, with an increment of 0.02); for each combination of α and ν, β was chosen to fix R_0 at the desired value in the no intervention case.

Performing analogous methods as for the other interventions, per α-ν pair we ascertained the vaccine uptake that maximised the intervention unit threshold value. We also explored the model's sensitivity to the vaccine efficacy, in this case modulating the risk of a breakthrough infection by a vaccinated, severe infection case.

4.3 Summary of findings

Across the collection of results, we report no difference in the outcomes between the (solely) infection blocking vaccine and the infection blocking vaccine where only severe cases could cause breakthrough infections (Figs. D and E).

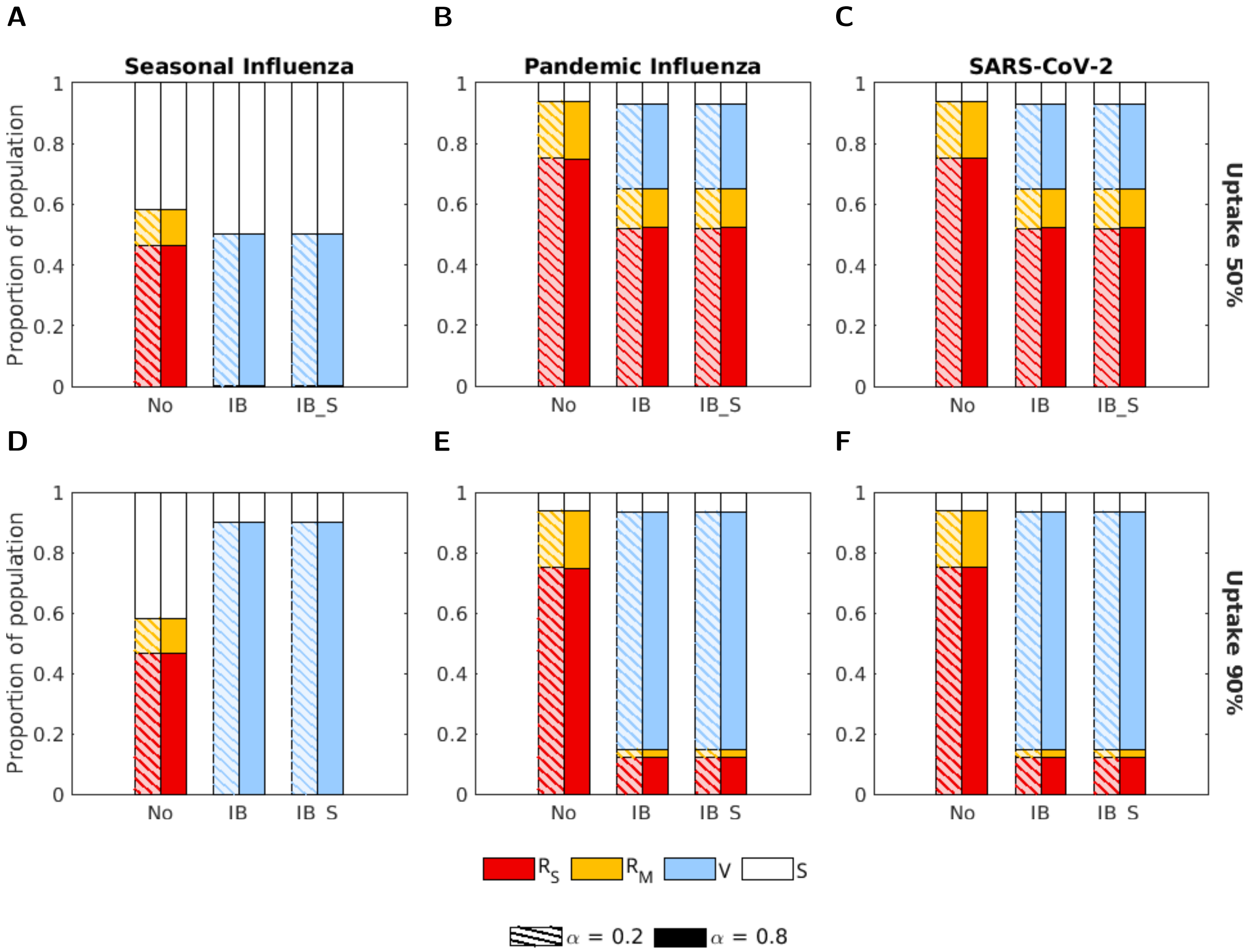

Figure D: Proportion of the population in each disease state at the end of the outbreak for each disease parameterisation given use of an infection blocking vaccine where only severe cases can cause breakthrough infections. The three groups of bars correspond to three intervention scenarios: no intervention (No), an infection blocking vaccine (IB) and an infection blocking vaccine for which only severe cases can cause breakthrough infections (IB_S). The two bars in each group correspond to symptom propagation strengths of $\alpha = 0.2$ (left bar, hatched lines) and $\alpha = 0.8$ (right bar, solid fill). Bar shading corresponds to disease status: red - recovered from severe infection (R_S); yellow - recovered from mild infection (R_M); blue - susceptible and vaccinated (V); white - susceptible and not vaccinated (S). The two rows correspond to two vaccine uptake levels: **(a-c)** 50%; **(d-f)** 90%. Columns correspond to differing disease parameterisations: **(a,d)** seasonal influenza; **(b,e)** pandemic influenza; **(c,f)** SARS-CoV-2. We fixed the vaccine efficacy at 70% and all other parameters were as given in Table 1, with ν chosen to fix the proportion of cases that were severe equal to 0.8.

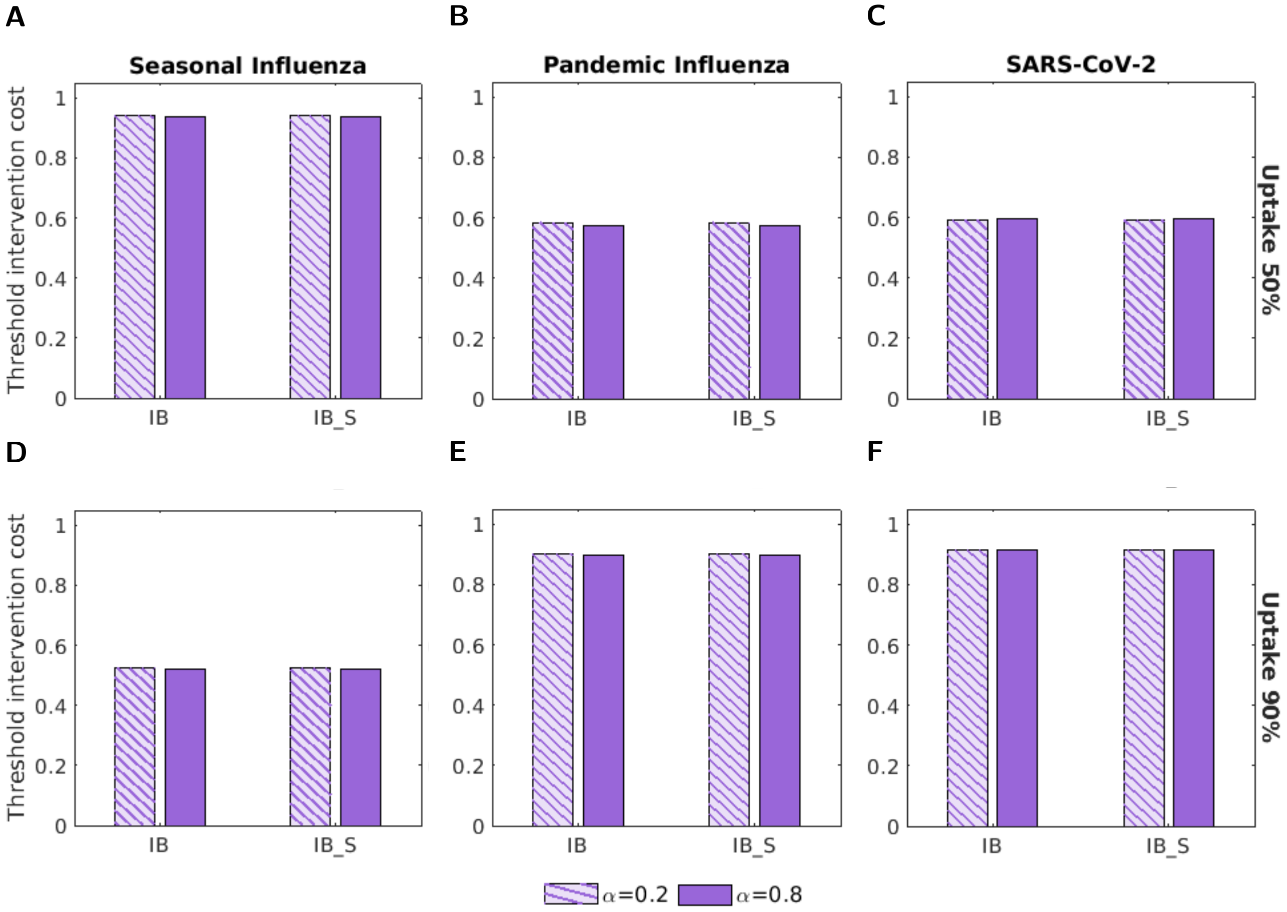

Figure E: The threshold unit intervention cost, under each disease parameterisation, for an infection blocking vaccine for which only severe cases can cause breakthrough infections. We normalise the threshold unit intervention cost by the highest absolute threshold unit intervention cost attained across the range of tested vaccine uptake values. The two groups of bars correspond to an infection blocking vaccine (IB) and an infection blocking vaccine for which only severe cases can cause breakthrough infections (IB_S). The two bars in each group correspond to symptom propagation strengths of $\alpha = 0.2$ (left bar, hatched lines) and $\alpha = 0.8$ (right bar, solid fill). The two rows correspond to two vaccine uptake levels: **(a-c)** 50%; **(d-f)** 90%. Columns correspond to differing disease parameterisations: **(a,d)** seasonal influenza; **(b,e)** pandemic influenza; **(c,f)** SARS-CoV-2. The efficacy was fixed at 70% and all other parameters were as given in Table 1 with ν chosen to fix the proportion of cases that were severe equal to 0.8.

5 Additional epidemiological outcomes

5.1 Final outbreak size and peak prevalence

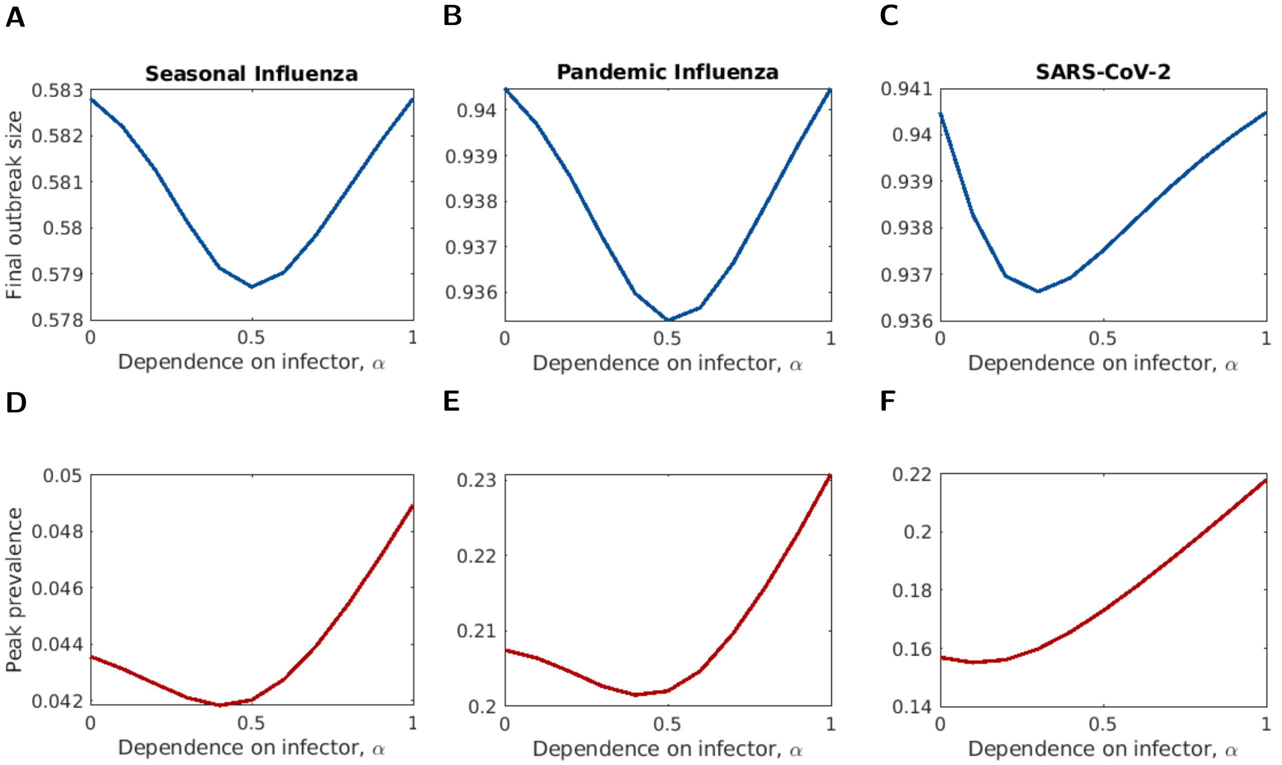

Figure F: The final outbreak size and peak prevalence for three parameter sets whilst varying the dependence on the infector's symptom severity, α. The top row shows the total proportion of the population infected across both severity classes. The bottom row shows the proportion of the population infected across both severity classes at the peak of the outbreak. Note that the y-axis scale varies between plots in the same row.

5.2 Temporal intervention plots

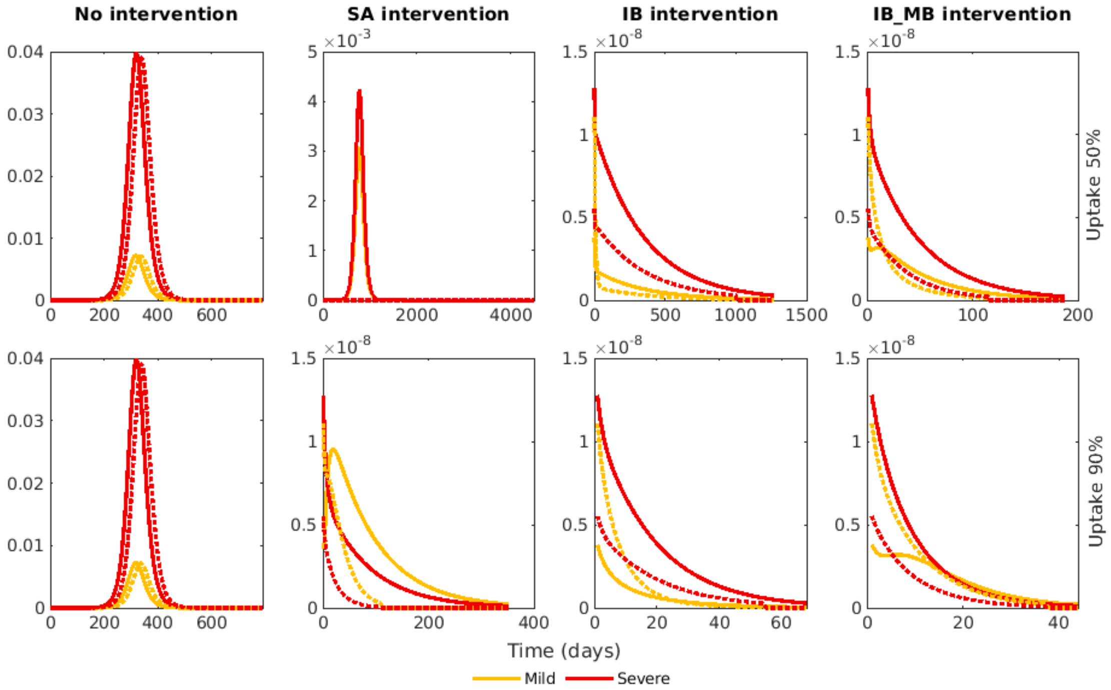

Figure G: Temporal profiles of infection prevalence in the absence of interventions and our three types of intervention for a pathogen with seasonal influenza-like attributes. The four columns correspond to four intervention scenarios: no intervention, a symptom-attenuating vaccine, an infection-blocking vaccine and an infection-blocking vaccine that only admits mild breakthrough infections. The rows correspond to two vaccine uptake levels, 50% and 90%. We fixed the vaccine efficacy at 70%. The line colour denotes the disease severity, with the pink line showing the proportion of the population who were infectious with mild disease and the red line showing the proportion of the population who were infectious with severe disease. The line style denotes the value of α, with the solid lines corresponding to $\alpha = 0.2$ and the dotted lines corresponding to $\alpha = 0.8$. Throughout, ν was chosen to fix the proportion of cases that were severe equal to 0.8. Note the differing scales on the y-axis.

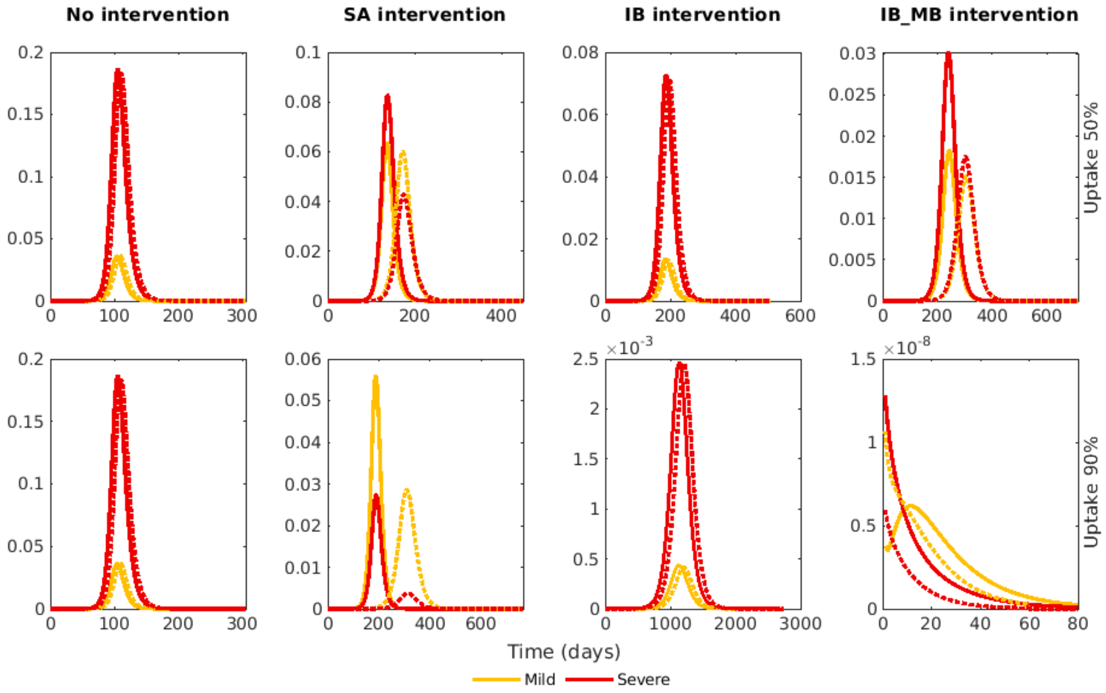

Figure H: Temporal profiles of infection prevalence in the absence of interventions and our three types of intervention for a pathogen with pandemic influenza-like attributes. The four columns correspond to four intervention scenarios: no intervention, a symptom-attenuating vaccine, an infection-blocking vaccine and an infection-blocking vaccine that only admits mild breakthrough infections. The rows correspond to two vaccine uptake levels, 50% and 90%. We fixed the vaccine efficacy at 70%. The line colour denotes the disease severity, with the pink line showing the proportion of the population who were infectious with mild disease and the red line showing the proportion of the population who were infectious with severe disease. The line style denotes the value of α, with the solid lines corresponding to $\alpha = 0.2$ and the dotted lines corresponding to $\alpha = 0.8$. Throughout, ν was chosen to fix the proportion of cases that were severe equal to 0.8. Note the differing scales on the y-axis.

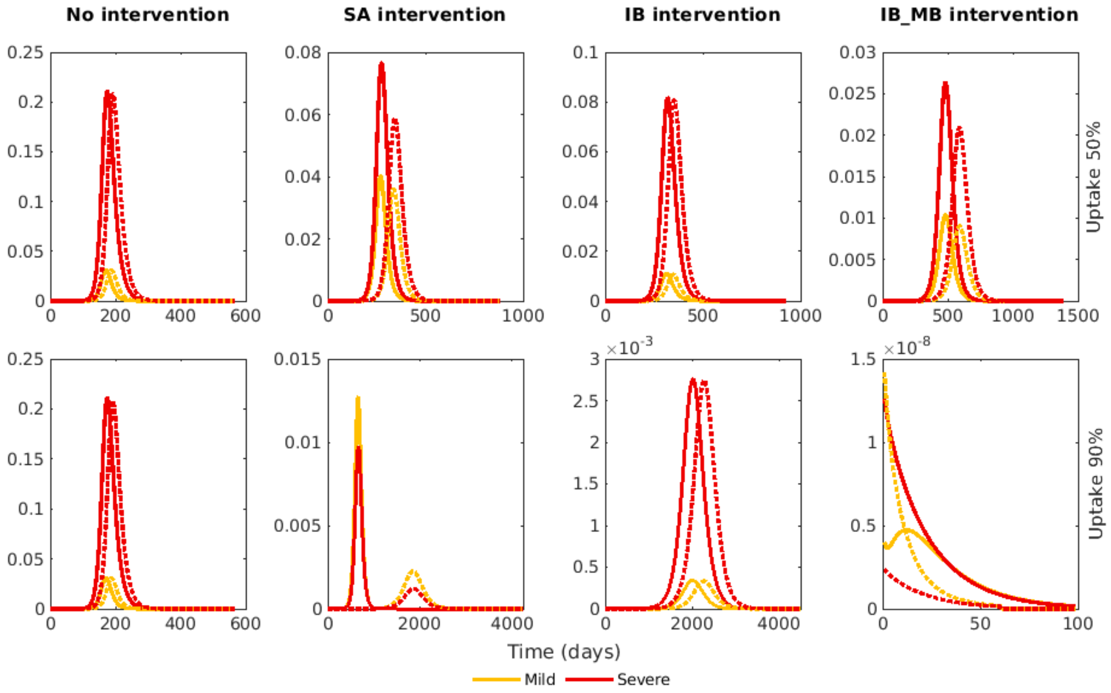

Figure I: Temporal profiles of infection prevalence in the absence of interventions and our three types of intervention for a pathogen with SARS-CoV-2-like attributes. The four columns correspond to four intervention scenarios: no intervention, a symptom-attenuating vaccine, an infection-blocking vaccine and an infection-blocking vaccine that only admits mild breakthrough infections. The rows correspond to two vaccine uptake levels, 50% and 90%. We fixed the vaccine efficacy at 70%. The line colour denotes the disease severity, with the pink line showing the proportion of the population who were infectious with mild disease and the red line showing the proportion of the population who were infectious with severe disease. The line style denotes the value of α, with the solid lines corresponding to $\alpha = 0.2$ and the dotted lines corresponding to $\alpha = 0.8$. Throughout, ν was chosen to fix the proportion of cases that were severe equal to 0.8. Note the differing scales on the y-axis.

5.3 Additional intervention bar plots

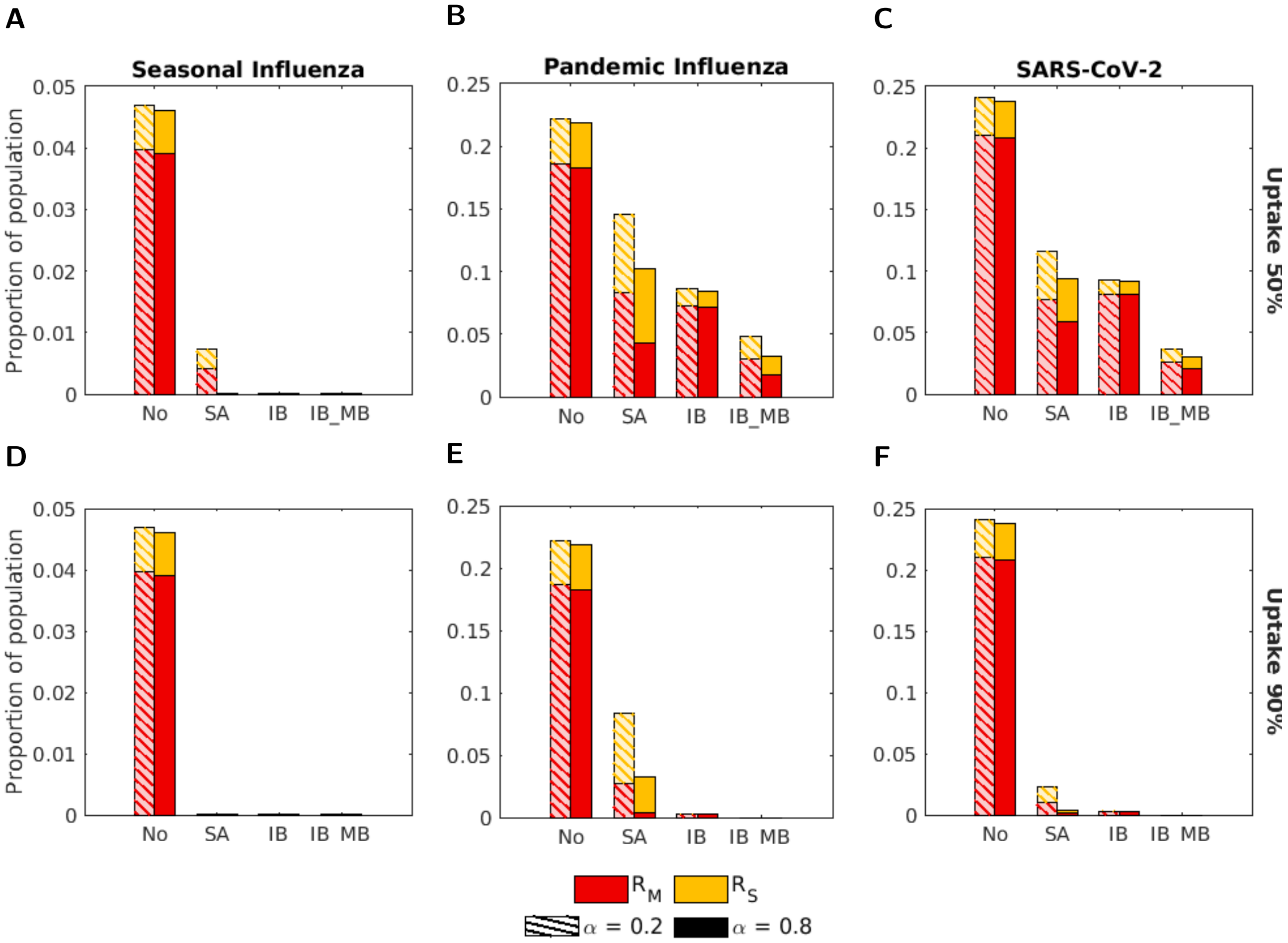

Figure J: Impact of disease parameterisation, action of intervention and intervention efficacy on peak infection prevalence. The four groups of bars correspond to four intervention scenarios: no intervention (No), a symptom attenuating vaccine (SA), an infection blocking vaccine (IB) and an infection blocking vaccine which only admits mild breakthrough infections (IB_MB). The two bars in each group correspond to symptom propagation strengths of $\alpha = 0.2$ (left bar, hatched lines) and $\alpha = 0.8$ (right bar, solid fill). Bar heights correspond to the peak in infection prevalence, with red and yellow bars representing severe and mild cases, respectively. The two rows correspond to two levels of vaccine uptake: **(a-c)** 50%; **(d-f)** 90%. The columns corresponded to one of the disease parameterisations: **(a,d)** seasonal influenza; **(b,e)** pandemic influenza; **(c,f)** SARS-CoV-2. We fixed the vaccine efficacy at 70%. All other parameters were as given in Table 1 with ν chosen to fix the proportion of cases that were severe equal to 0.8.

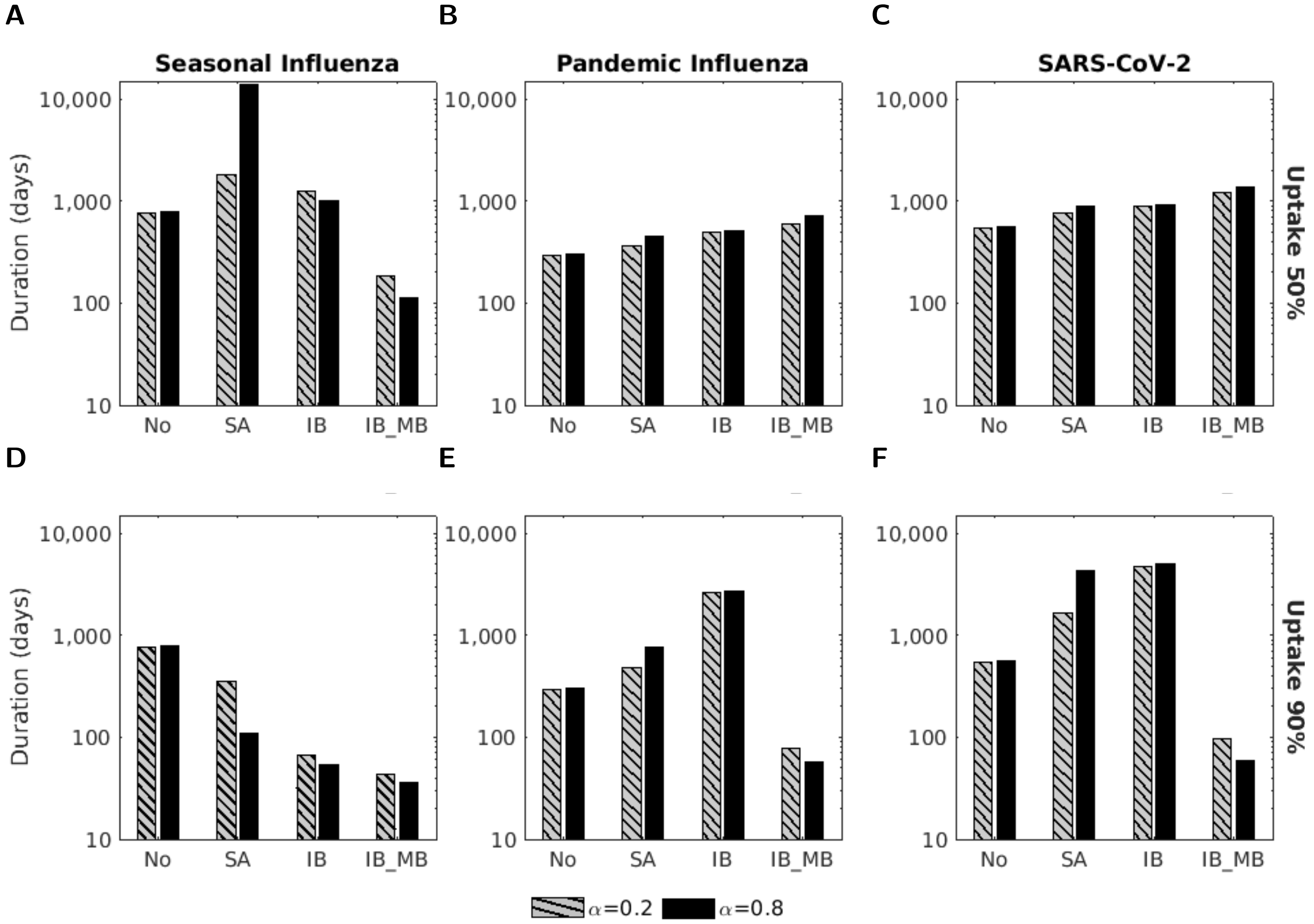

Figure K: Impact of disease parameterisation, action of intervention and intervention efficacy on outbreak duration. In each panel, the four groups of bars correspond to four intervention scenarios: no intervention (No), a symptom attenuating vaccine (SA), an infection blocking vaccine (IB) and an infection blocking vaccine which only admits mild breakthrough infections (IB_MB). The two bars in each group correspond to symptom propagation strengths of $\alpha = 0.2$ (left bar, hatched lines) and $\alpha = 0.8$ (right bar, solid fill). Bar heights correspond to outbreak duration in days. The two rows correspond to two levels of vaccine uptake: **(a-c)** 50%; **(d-f)** 90%. The columns corresponded to one of the disease parameterisations: **(a,d)** seasonal influenza; **(b,e)** pandemic influenza; **(c,f)** SARS-CoV-2. We fixed the vaccine efficacy at 70%. All other parameters were as given in Table 1 with ν chosen to fix the proportion of cases that were severe equal to 0.8.

5.4 Outcomes conditional on $\nu = 0.2$

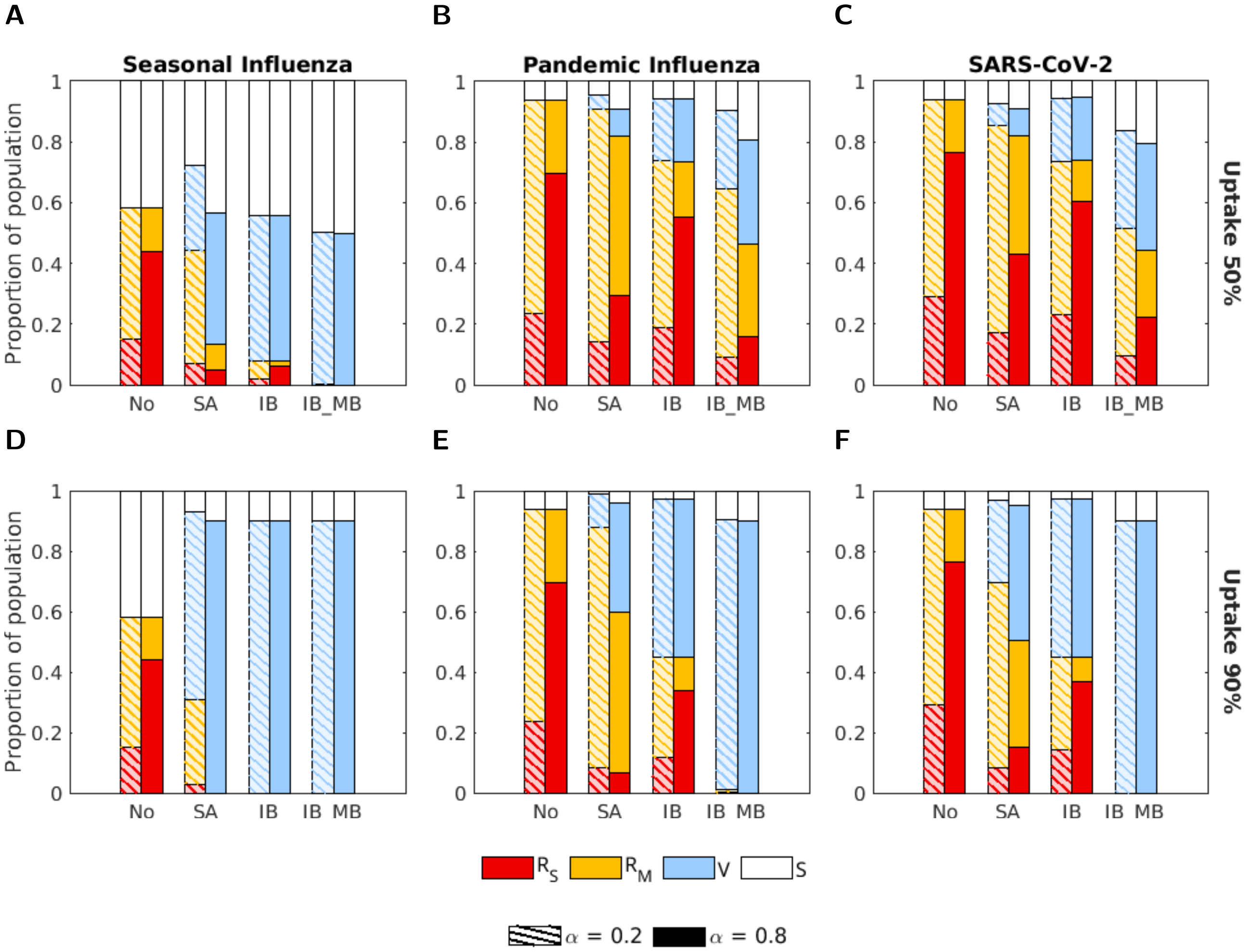

Figure L: End of outbreak disease status composition under an alternative baseline severity assumption. Analogous to Fig 5 in the main manuscript, except here we used a fixed value of $\nu = 0.2$ (instead of calibrating the value of ν to attain 80% of cases being severe in the no intervention scenario). In each panel, the four groups of bars correspond to four intervention scenarios: no intervention (No), a symptom attenuating vaccine (SA), an infection blocking vaccine (IB) and an infection blocking vaccine which only admits mild breakthrough infections (IB_MB). The two bars in each group correspond to symptom propagation strengths of $\alpha = 0.2$ (left bar, hatched lines) and $\alpha = 0.8$ (right bar, solid fill). The shading of the bar corresponds to four disease status compartments: red - recovered from severe infection (R_S); yellow - recovered from mild infection (R_M); blue - susceptible and vaccinated (V); white - susceptible and not vaccinated (S). The two rows correspond to two uptake levels: **(a-c)** 50%; **(d-f)** 90%. Columns correspond to differing disease parameterisations: **(a,d)** seasonal influenza; **(b,e)** pandemic influenza; **(c,f)** SARS-CoV-2. We fixed the vaccine efficacy at 70% and all other parameters were as given in Table 1.

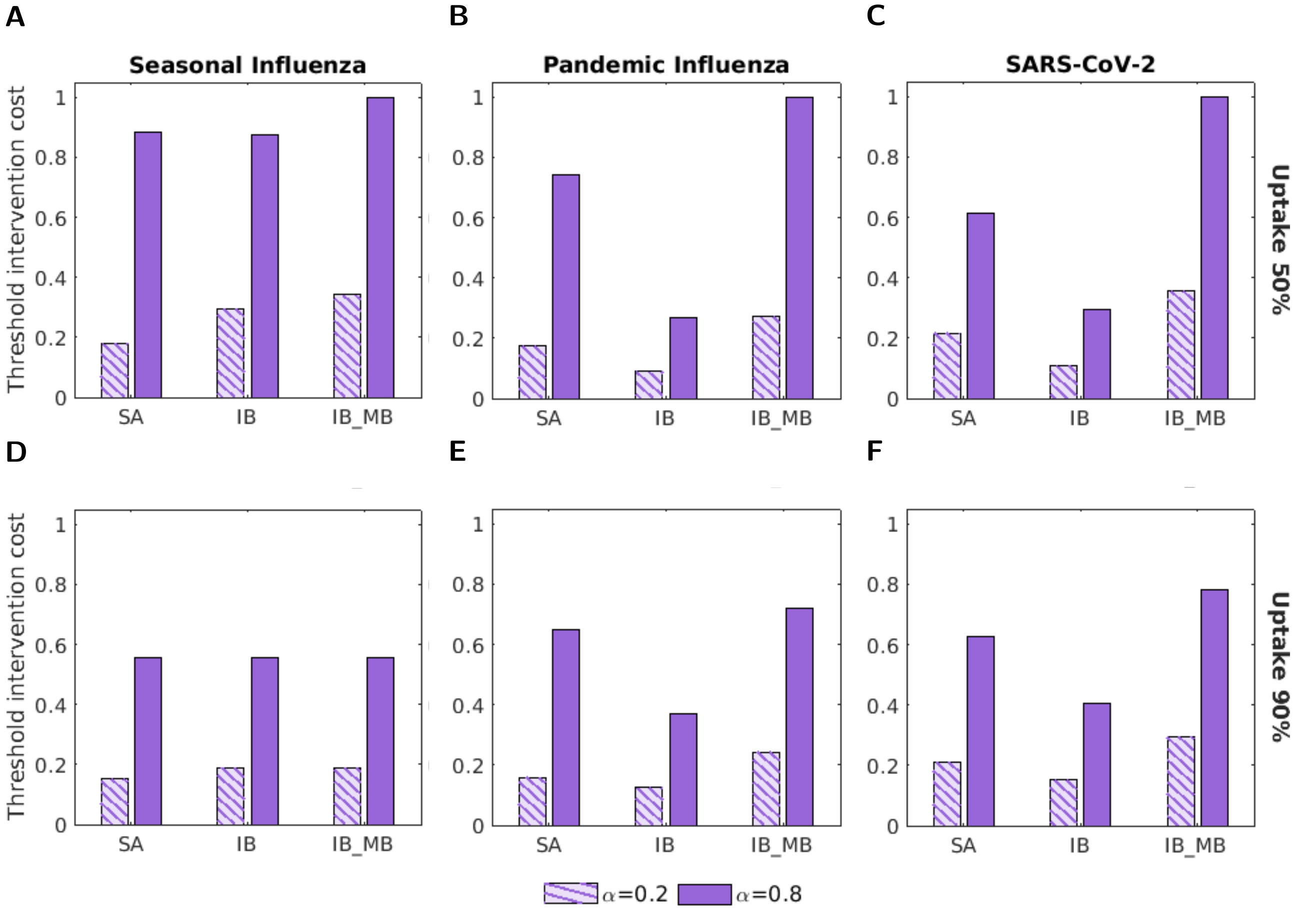

Figure M: Threshold unit intervention costs for three interventions and three disease parameterisations under an alternative value of ν. Analogous to Fig 7 in the main manuscript, except here we used a fixed value of $\nu = 0.2$ (instead of calibrating the value of ν to attain 80% of cases being severe in the no intervention scenario). In all panels, we normalised threshold unit intervention costs by the highest absolute threshold unit intervention cost attained across the range of tested intervention uptake values. The three groups of bars correspond to three interventions: a symptom attenuating vaccine (SA), an infection blocking vaccine (IB) and an infection blocking vaccine that only admits mild breakthrough infections (IB_MB). The two bars in each group correspond to symptom propagation strengths of $\alpha = 0.2$ (left bar, hatched lines) and $\alpha = 0.8$ (right bar, solid fill). The two rows correspond to two vaccine uptake levels: **(a-c)** 50%; **(d-f)** 90%. Columns correspond to differing disease parameterisations: **(a,d)** seasonal influenza; **(b,e)** pandemic influenza; **(c,f)** SARS-CoV-2. Vaccine efficacies were fixed at 70% and all other parameters were as given in Table 1.

6 Sensitivity to discounting

Whilst in the main text we used a discount rate of 3.5% on monetary costs and QALY losses, here we performed similar health economic analyses with no discounting applied (i.e. a discounting rate of 0%).

Overall, when applying no discounting, we found quantitatively similar findings (as in the main manuscript) for the relative threshold unit intervention costs for each combination of disease parameterisation and action of intervention (Figs. N and O).

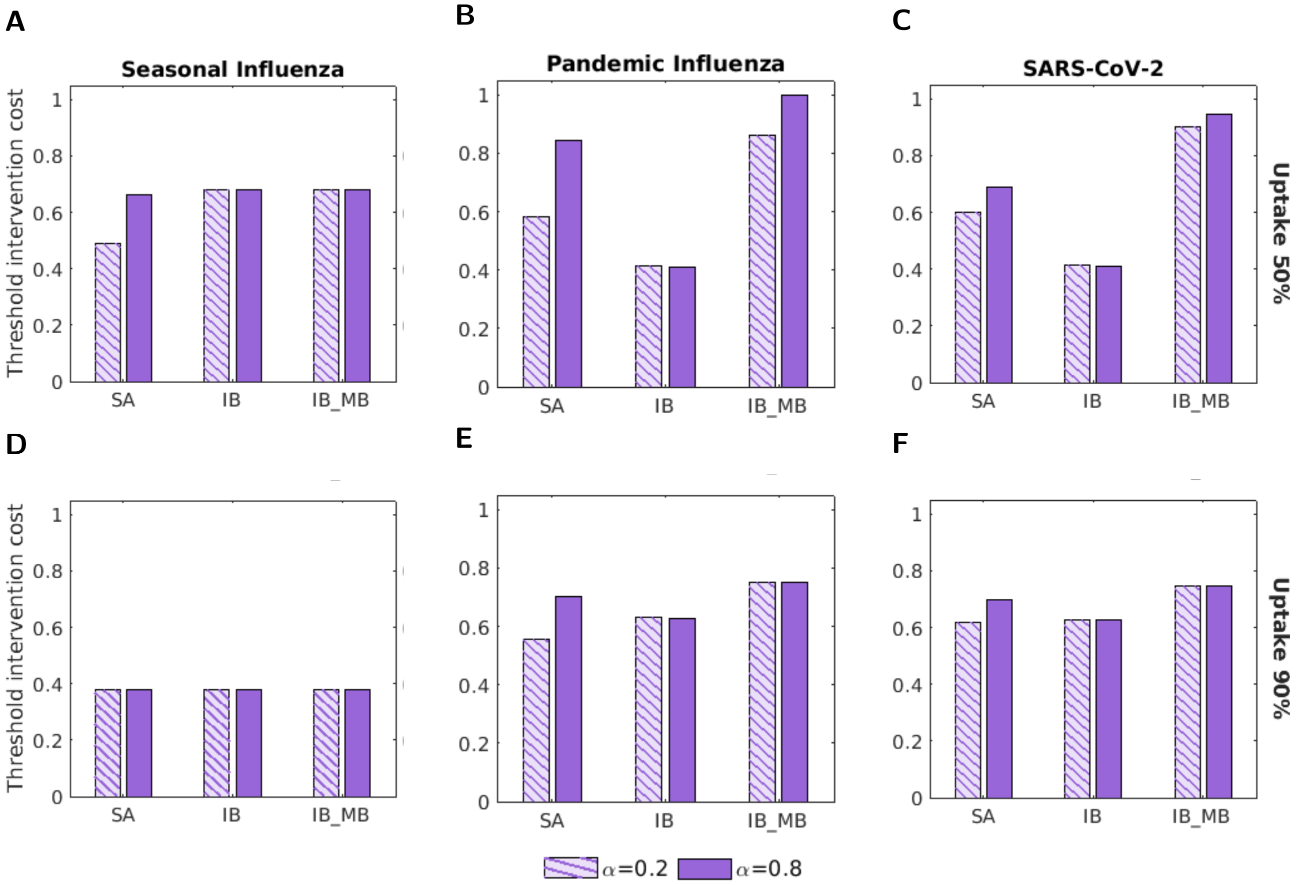

Figure N: The threshold unit intervention costs for the three interventions and three disease parameterisations with no discounting applied. Analogous to Fig 7 in the main manuscript, but using a discount rate of 0% (whereas in the main text we used a discount rate of 3.5%). In all panels, we normalised threshold unit intervention costs by the highest absolute threshold unit intervention cost attained across the range of tested intervention uptake values. The three groups of bars correspond to three interventions: a symptom attenuating vaccine(SA), an infection blocking vaccine (IB) and an infection blocking vaccine that only admits mild breakthrough infections (IB_MB). The two bars in each group correspond to symptom propagation strengths of $\alpha = 0.2$ (left bar, hatched lines) and $\alpha = 0.8$ (right bar, solid fill). The two rows correspond to two vaccine uptake levels: **(a-c)** 50%; **(d-f)** 90%. Columns correspond to differing disease parameterisations: **(a,d)** seasonal influenza; **(b,e)** pandemic influenza; **(c,f)** SARS-CoV-2. Vaccine efficacies were fixed at 70% and all other parameters were as given in Table 1, with ν chosen to fix the proportion of cases that were severe equal to 0.8. The results shown here are qualitatively similar to those obtained when using 3.5% discounting.

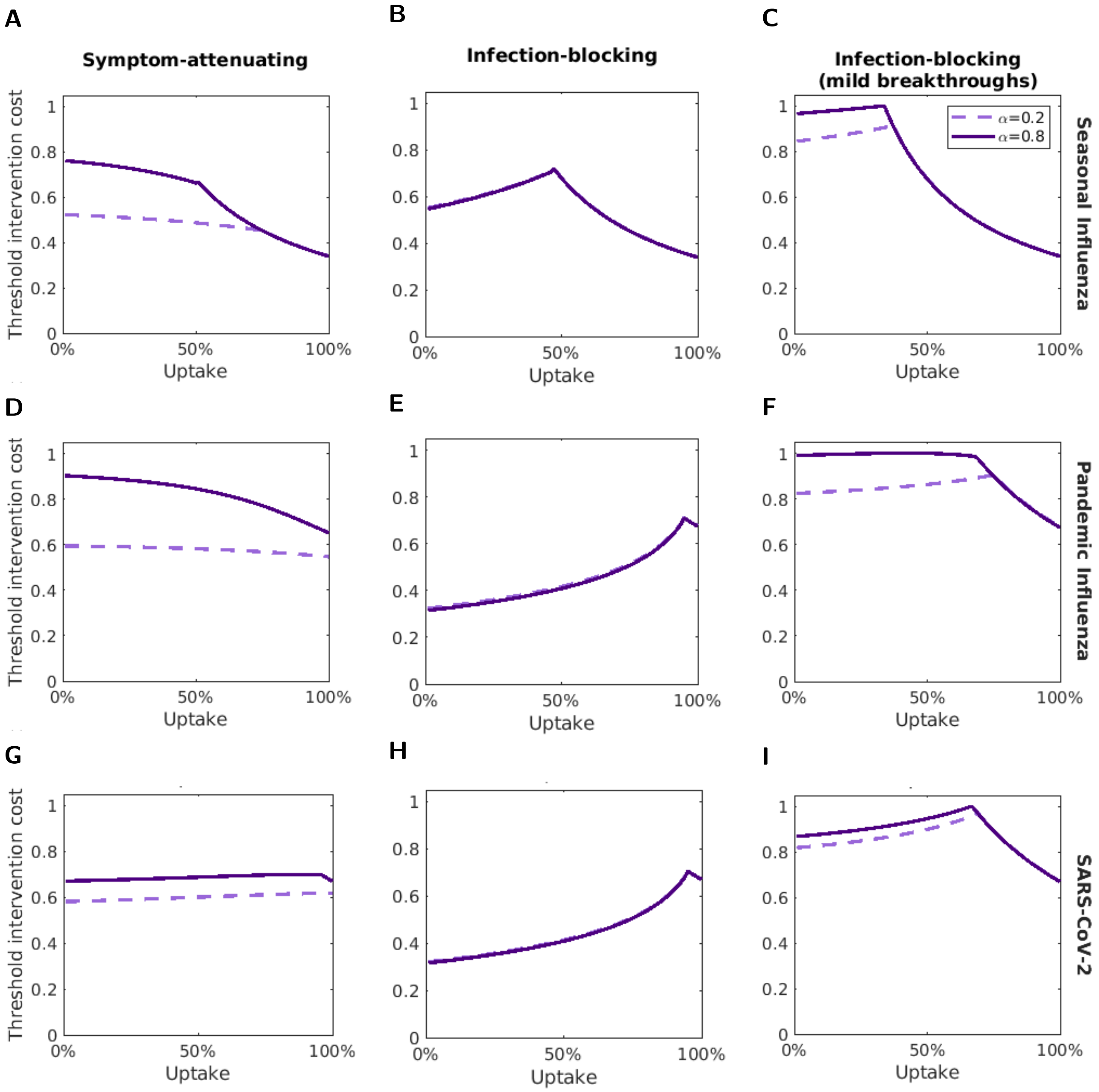

Figure O: Variation of the threshold unit intervention cost with intervention uptake, with no discounting being applied. Analogous to Fig 8 in the main manuscript, but using a discount rate of 0% (whereas in the main text we used a discount rate of 3.5%). We normalised threshold unit intervention costs for each disease parameterisation; the normalisation constant was the highest absolute threshold unit intervention cost attained for the respective disease parameterisation across the range of tested intervention uptake values. The three rows correspond to different disease parameterisations: **(a-c)** seasonal influenza; **(d-f)** pandemic influenza; **(g-i)** SARS-CoV-2. The three columns correspond to three interventions: **(a,d,g)** a symptom-attenuating vaccine (SA), **(b,e,h)** an infection-blocking vaccine (IB) and **(c,f,i)** an infection-blocking vaccine that only admits mild breakthrough infections (IB_MB). The two lines correspond to two symptom propagation strengths; the dashed, light purple line corresponds to $\alpha = 0.2$ and the solid, dark purple line corresponds to $\alpha = 0.8$. We fixed vaccine efficacies at 70% and all other parameters values were as given in Table 1, with ν chosen to fix the proportion of cases that were severe equal to 0.8.

7 Sensitivity to intervention efficacy

In the main text we considered vaccination interventions with an efficacy of 70%. Here we performed the same analysis with two alternative vaccine efficacy values: 50% and 90%.

Overall, we found qualitatively similar results for all three vaccine efficacy values. As expected, generally fewer infections were prevented when the efficacy was 50% (Fig. P) and more infections were prevented when the efficacy was 90% (Fig. T). However, the patterns between intervention types and the two α values were consistent across the three levels of vaccine efficacy.

We did reveal that the relative effectiveness of a symptom attenuating and infection blocking intervention varied with the efficacy value. At 50% efficacy, a symptom-attenuating vaccine was more effective for a larger proportion of the parameter space, as indicated by the increase in areas shaded in red (Fig. Q). At 90% efficacy, an infection-blocking vaccine was more effective for a larger proportion of the parameter space, as indicated by the increase in areas shaded in blue (Fig. U).

Patterns in threshold unit intervention cost were similar across the three efficacy levels, although the relative cost between the three intervention types varied. At 50% efficacy, the infection blocking vaccine was generally much less cost-effective than the other two types (Figs. R and S), whereas at 90% efficacy the threshold unit intervention cost was more consistent across intervention types (Figs. V and W).

7.1 50% efficacy

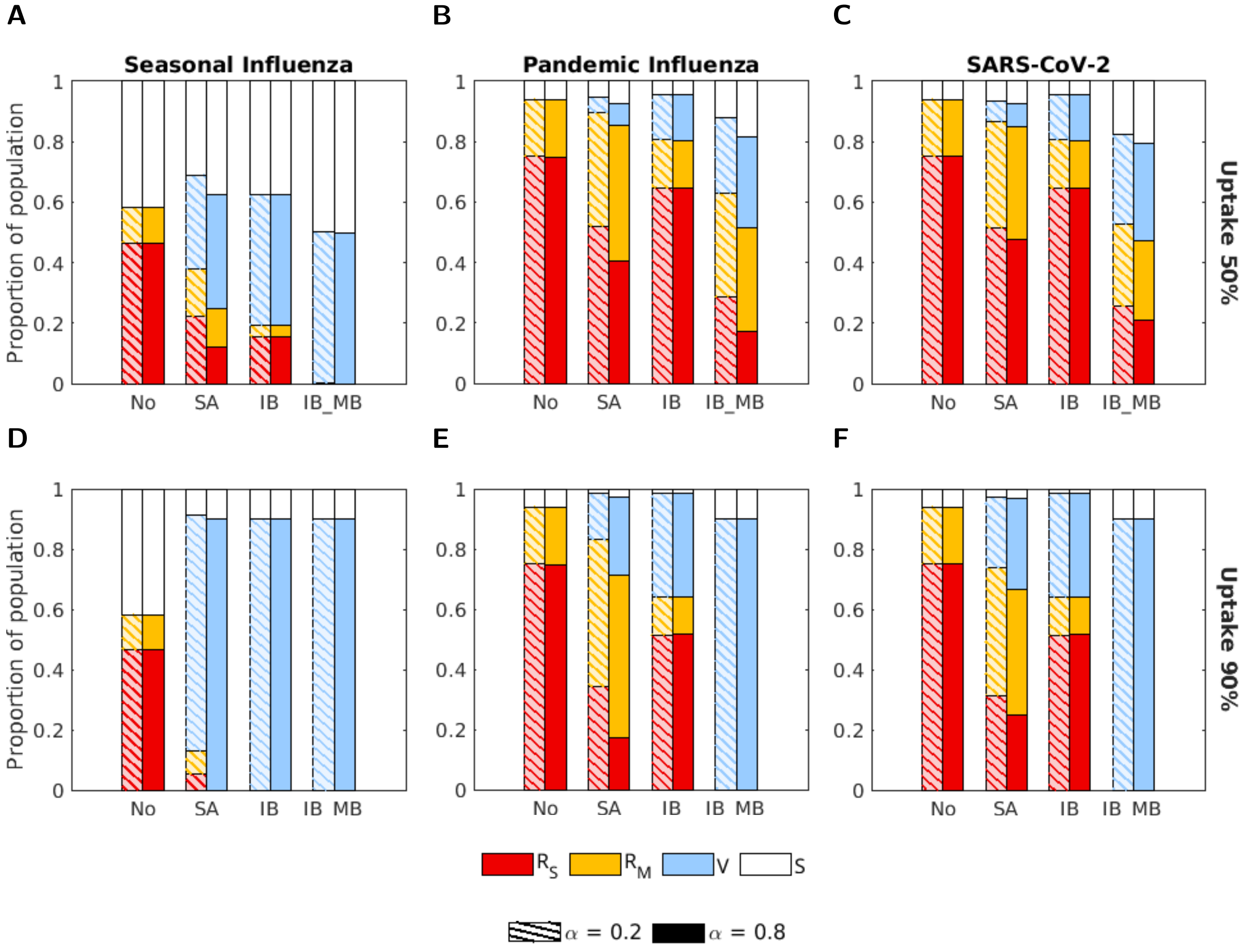

Figure P: The proportion of the population in each disease state at the end of the outbreak for the four intervention scenarios (with an intervention efficacy of 50%) and three disease parameterisations. The four groups of bars correspond to four intervention scenarios: no intervention (No), a symptom attenuating vaccine (SA), an infection blocking vaccine (IB) and an infection blocking vaccine which only admits mild breakthrough infections (IB_MB). The two bars in each group correspond to symptom propagation strengths of $\alpha = 0.2$ (left bar, hatched lines) and $\alpha = 0.8$ (right bar, solid fill). Bar shading corresponds to disease status: red - recovered from severe infection (R_S); yellow - recovered from mild infection (R_M); blue - susceptible and vaccinated (V); white - susceptible and not vaccinated (S). The two rows correspond to two vaccine uptake levels: **(a-c)** 50%; **(d-f)** 90%. Columns correspond to differing disease parameterisations: **(a,d)** seasonal influenza; **(b,e)** pandemic influenza; **(c,f)** SARS-CoV-2. We fixed vaccination efficacies at 50% and all other parameters were as given in Table 1, with ν chosen to fix the proportion of cases that were severe equal to 0.8.

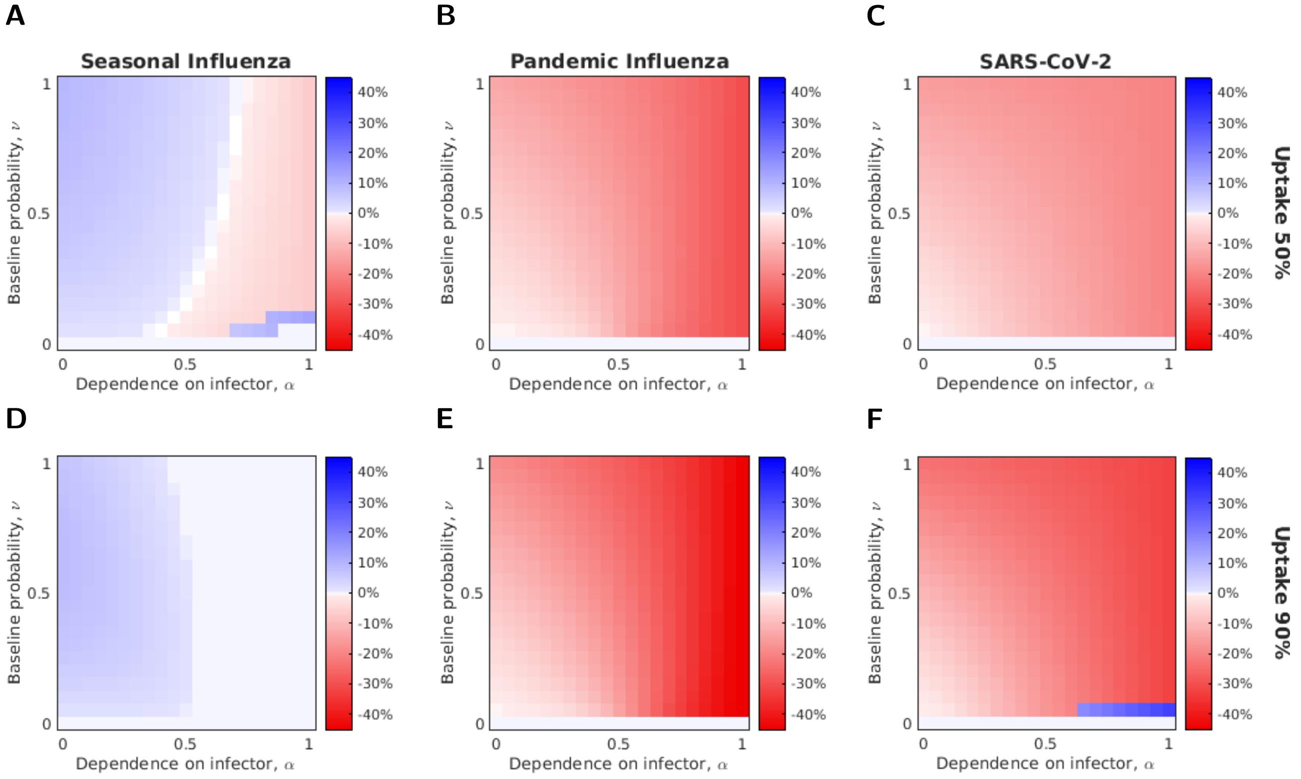

Figure Q: The relative effectiveness of a symptom attenuating and infection blocking vaccine with a fixed efficacy (50%) varies with α and ν. Each row corresponds to one of the two uptake levels: **(a-c)** 50%; **(d-f)** 90%. Each column corresponds to a different disease parameterisation: **(a,d)** seasonal influenza; **(b,e)** pandemic influenza; **(c,f)** SARS-CoV-2. Cell shading denotes (for that combination of α-ν value) the difference in the proportion of the population severely infected between when a symptom-attenuating intervention was used and when an infection-blocking intervention was used. The blue shaded cells shows parameter combinations where the infection blocking intervention was more effective at preventing infections, whilst the red shaded cells shows parameter combinations where symptom attenuation was more effective.

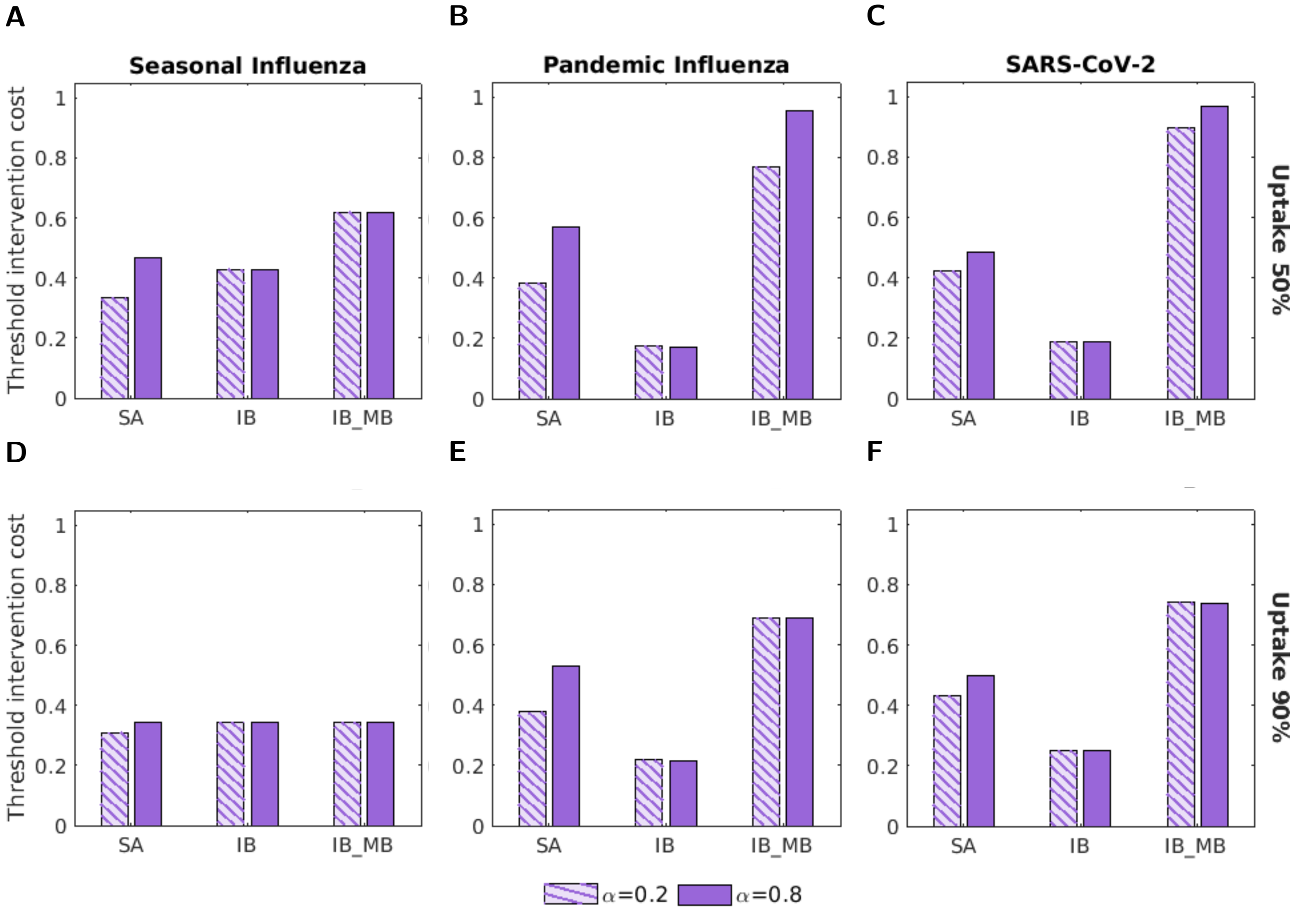

Figure R: The threshold unit intervention cost for the three vaccine interventions and each disease parameterisation with a vaccine efficacy of 50%. In all panels, we normalised threshold unit intervention cost by the highest absolute threshold unit intervention cost attained across the range of tested intervention uptake values. The three groups of bars correspond to three interventions: a symptom attenuating vaccine (SA), an infection blocking vaccine (IB) and an infection blocking vaccine that only admits mild breakthrough infections (IB_MB). The two bars in each group correspond to symptom propagation strengths of $\alpha = 0.2$ (left bar, hatched lines) and $\alpha = 0.8$ (right bar, solid fill). The two rows correspond to two vaccine uptake levels: **(a-c)** 50%; **(d-f)** 90%. Columns correspond to differing disease parameterisations: **(a,d)** seasonal influenza; **(b,e)** pandemic influenza; **(c,f)** SARS-CoV-2. Vaccine efficacies were fixed at 50% and all other parameters were as given in Table 1 with ν chosen to fix the proportion of cases that were severe equal to 0.8.

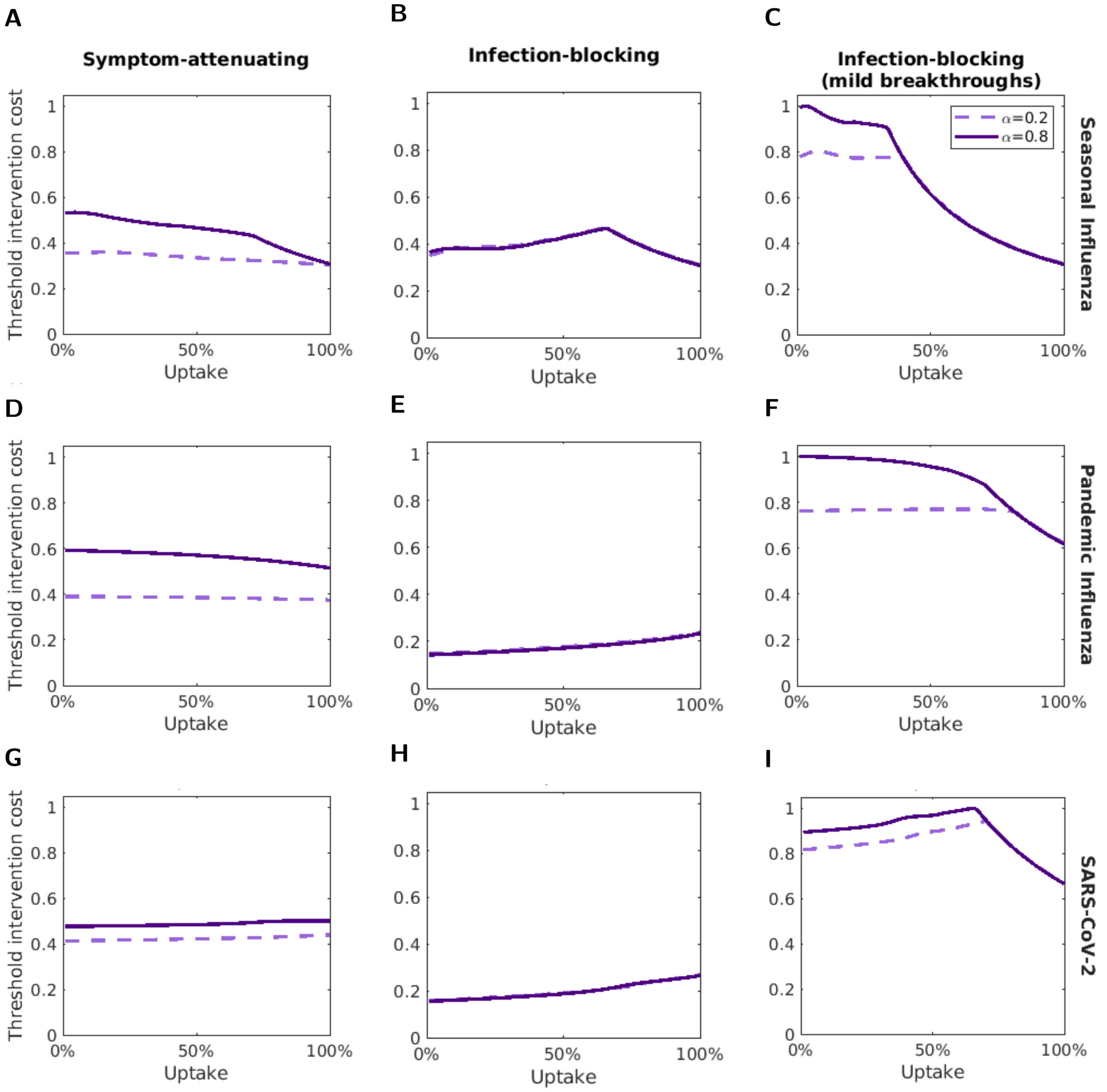

Figure S: The threshold unit intervention cost varies with vaccine uptake for a vaccine efficacy of 50%. We normalised threshold unit intervention costs for each disease parameterisation; the normalisation constant was the highest absolute threshold unit intervention cost attained for the respective disease parameterisation across the range of tested uptake values. The three rows correspond to the three different disease parameterisations: **(a-c)** seasonal influenza; **(d-f)** pandemic influenza; **(g-i)** SARS-CoV-2. The three columns correspond to three interventions: **(a,d,g)** a symptom-attenuating vaccine (SA), **(b,e,h)** an infection-blocking vaccine (IB) and **(c,f,i)** an infection-blocking vaccine that only admits mild breakthrough infections (IB_MB). The two lines correspond to two symptom propagation strengths; the dashed, light purple line corresponds to $\alpha = 0.2$ and the solid, dark purple line corresponds to $\alpha = 0.8$. We fixed the vaccine efficacies at 50% and all other parameters values were as given in Table 1, with ν chosen to fix the proportion of cases that were severe equal to 0.8.

7.2 90% efficacy

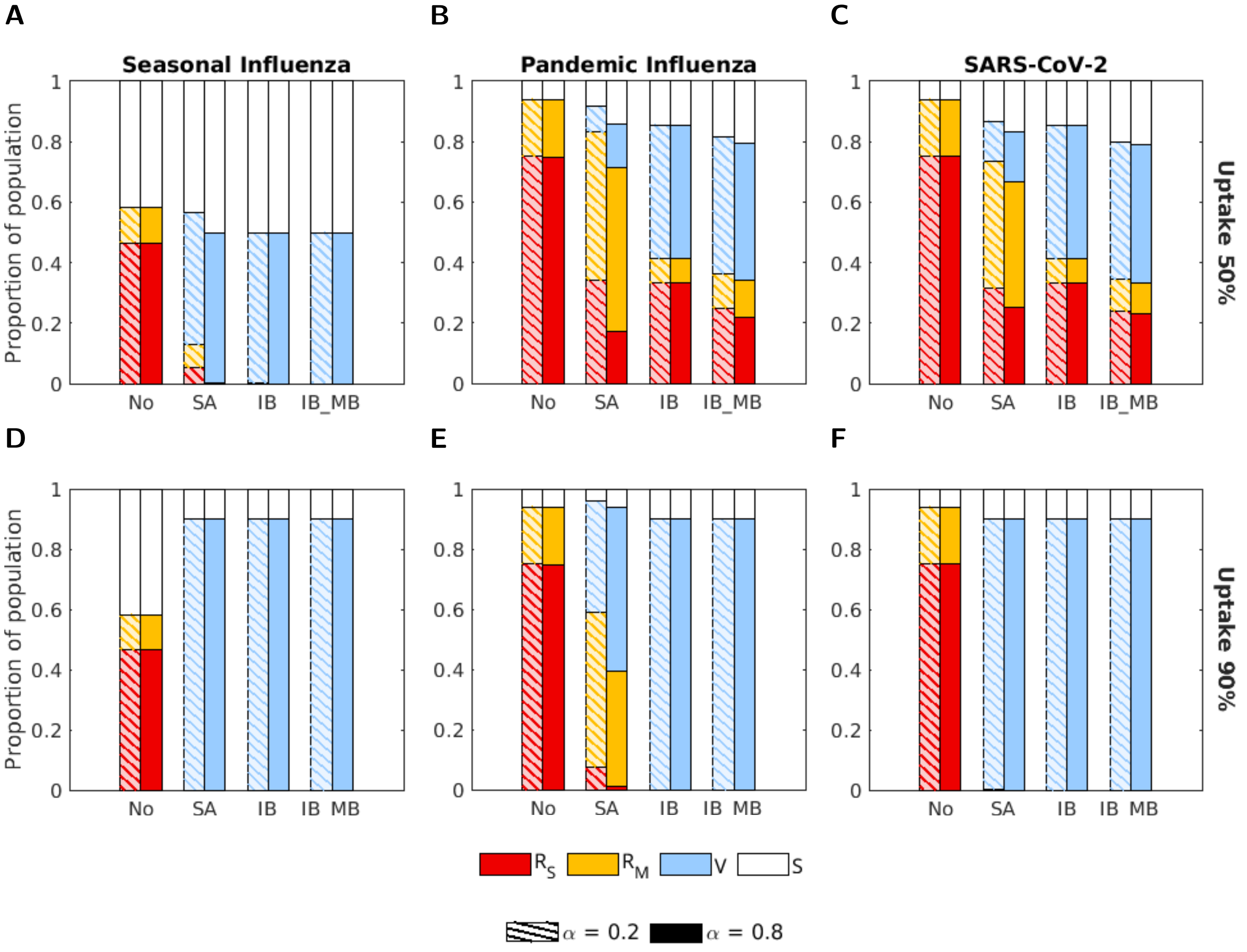

Figure T: The proportion of the population in each disease state at the end of the outbreak for the four intervention scenarios (with vaccine efficacies of 90%) and three disease parameterisations. The four groups of bars correspond to four intervention scenarios: no intervention (No), a symptom attenuating vaccine (SA), an infection blocking vaccine (IB) and an infection blocking vaccine that only admits mild breakthrough infections (IB_MB). The two bars in each group correspond to symptom propagation strengths of $\alpha = 0.2$ (left bar, hatched lines) and $\alpha = 0.8$ (right bar, solid fill). Bar shading corresponds to disease status: red - recovered from severe infection (R_S); yellow - recovered from mild infection (R_M); blue - susceptible and vaccinated (V); white - susceptible and not vaccinated (S). The two rows correspond to two vaccine uptake levels: **(a-c)** 50%; **(d-f)** 90%. Columns correspond to differing disease parameterisations: **(a,d)** seasonal influenza; **(b,e)** pandemic influenza; **(c,f)** SARS-CoV-2. We fixed the vaccine efficacies at 90% and all other parameters were as given in Table 1, with ν chosen to fix the proportion of cases that were severe equal to 0.8.

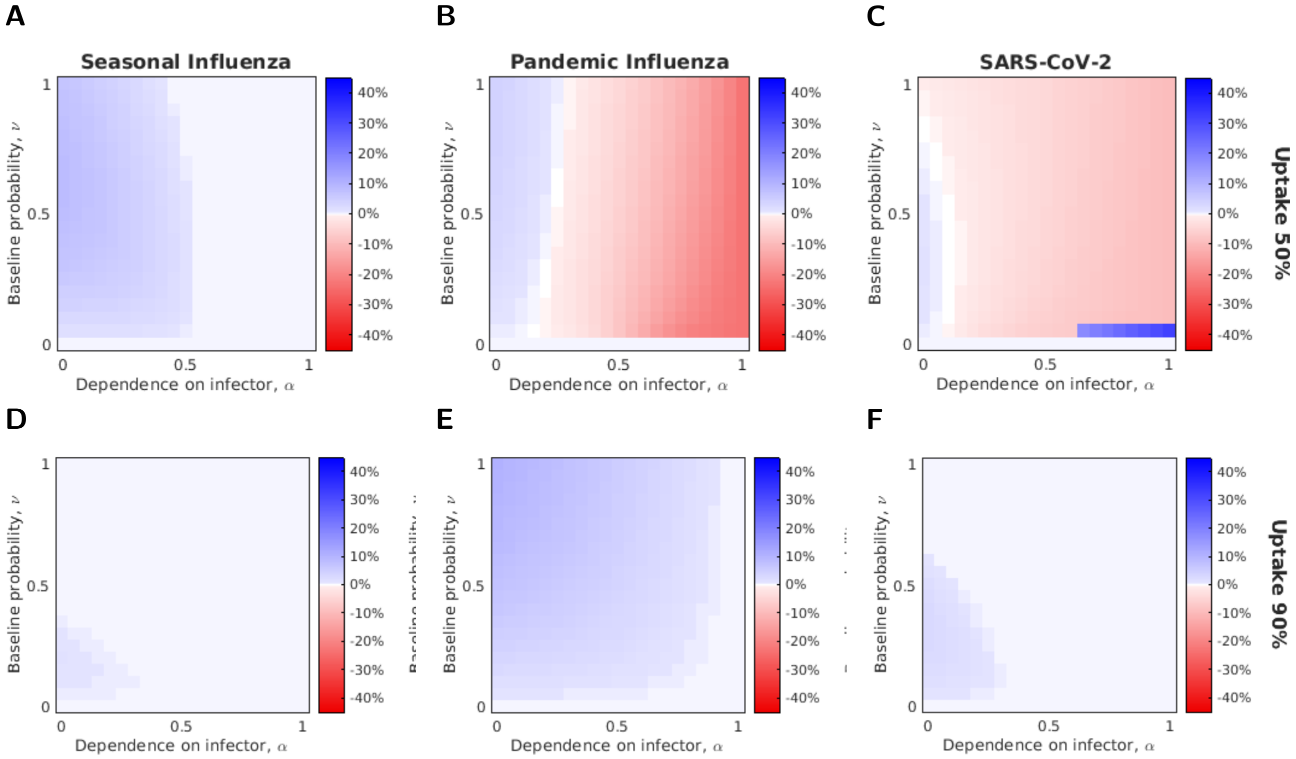

Figure U: The relative effectiveness of a symptom attenuating and infection blocking intervention with a fixed efficacy (90%) varies with α and ν. Each row corresponds to one of the two uptake levels: **(a-c)** 50%; **(d-f)** 90%. Each column corresponds to a different disease parameterisation: **(a,d)** seasonal influenza; **(b,e)** pandemic influenza; **(c,f)** SARS-CoV-2. Cell shading denotes (for that combination of α-ν value) the difference in the proportion of the population severely infected between when a symptom-attenuating intervention was used and when an infection-blocking intervention was used. The blue shaded cells shows parameter combinations where the infection blocking intervention was more effective at preventing infections, whilst the red shaded cells shows parameter combinations where symptom attenuation was more effective.

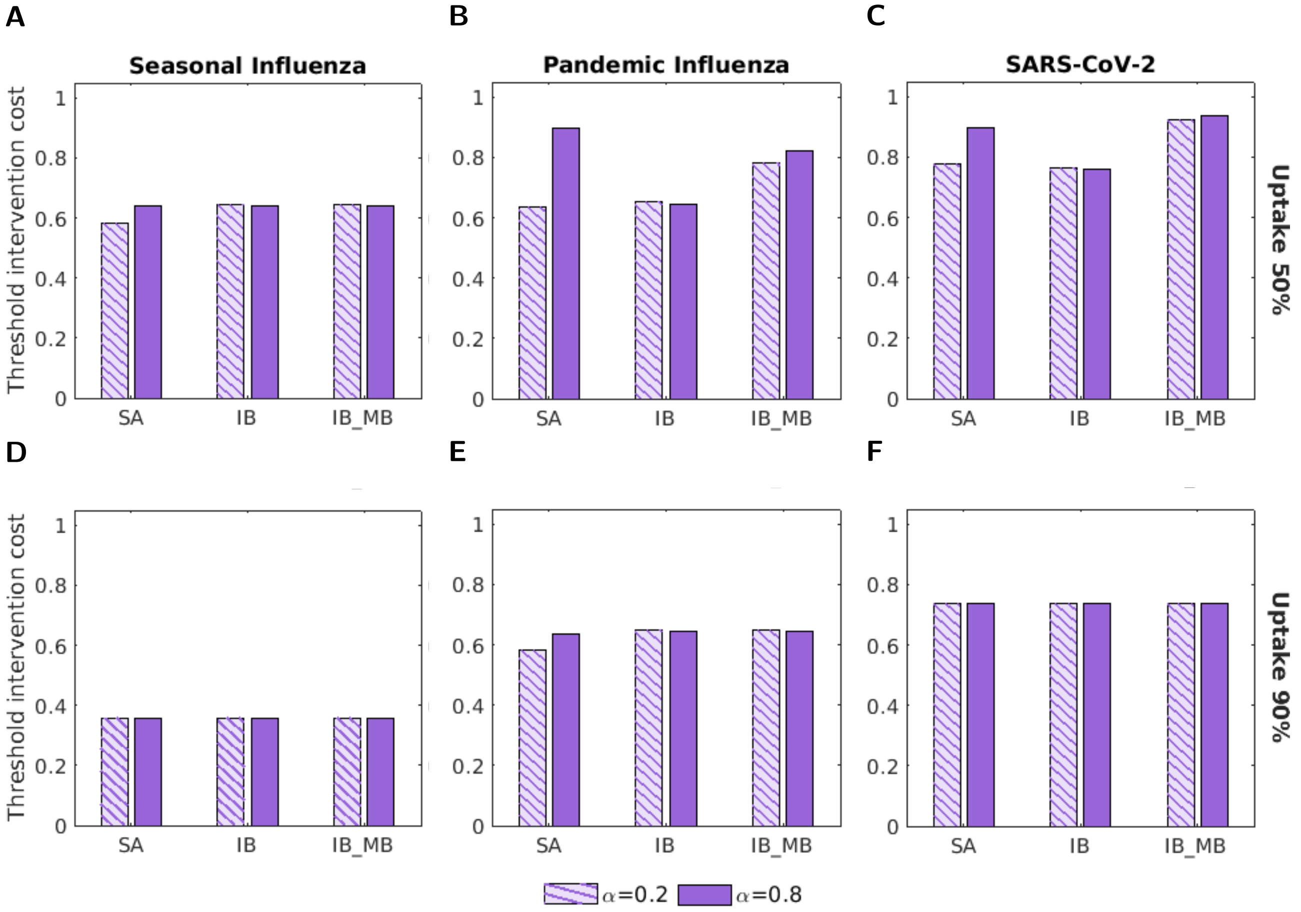

Figure V: The threshold unit intervention cost for the three vaccine interventions and each disease parameterisation with vaccine efficacies of 90%. In all panels, we normalised threshold unit intervention cost by the highest absolute threshold unit intervention cost attained across the range of tested intervention uptake values. The three groups of bars correspond to three interventions: a symptom attenuating vaccine (SA), an infection blocking vaccine (IB) and an infection blocking vaccine which only admits mild breakthrough infections (IB_MB). The two bars in each group correspond to symptom propagation strengths of $\alpha = 0.2$ (left bar, hatched lines) and $\alpha = 0.8$ (right bar, solid fill). The two rows correspond to two vaccine uptake levels: **(a-c)** 50%; **(d-f)** 90%. Columns correspond to differing disease parameterisations: **(a,d)** seasonal influenza; **(b,e)** pandemic influenza; **(c,f)** SARS-CoV-2. Vaccine efficacies were fixed at 90% and all other parameters were as given in Table 1 with ν chosen to fix the proportion of cases that were severe equal to 0.8.

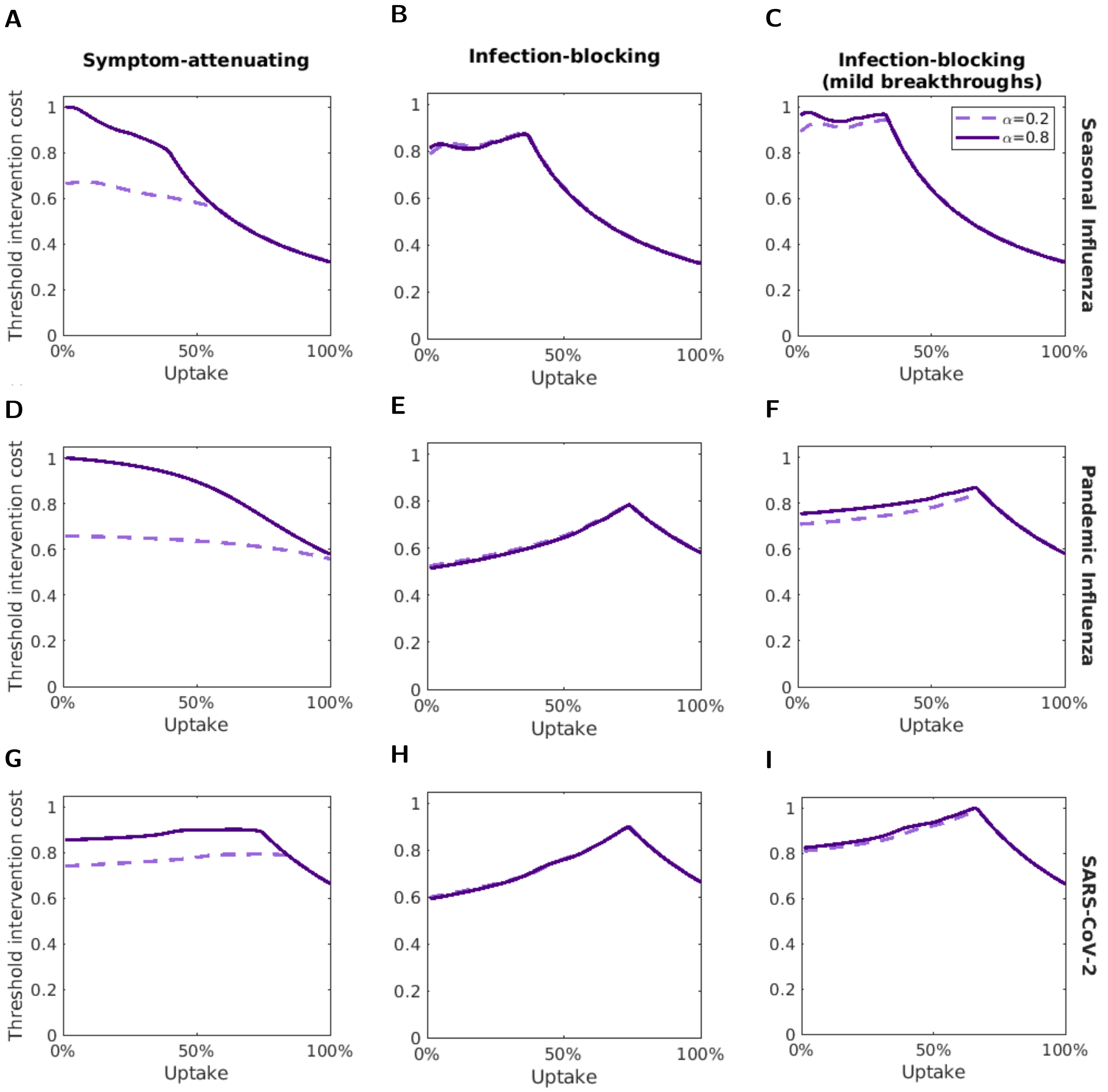

Figure W: Variation in the threshold unit intervention cost with vaccine uptake for vaccine efficacies of 90%. We normalised threshold unit intervention costs for each disease parameterisation; the normalisation constant was the highest absolute threshold unit intervention cost attained for the respective disease parameterisation across the range of tested vaccine uptake values. The three rows correspond to the three different disease parameterisations: **(a-c)** seasonal influenza; **(d-f)** pandemic influenza; **(g-i)** SARS-CoV-2. The three columns correspond to three interventions: **(a,d,g)** a symptom-attenuating vaccine (SA), **(b,e,h)** an infection-blocking vaccine (IB) and **(c,f,i)** an infection-blocking vaccine that only admits mild breakthrough infections (IB_MB). The two lines correspond to two symptom propagation strengths; the dashed, light purple line corresponds to $\alpha = 0.2$ and the solid, dark purple line corresponds to $\alpha = 0.8$. We fixed the vaccine efficacies at 90% and all other parameters values were as given in Table 1, with ν chosen to fix the proportion of cases that were severe equal to 0.8.

8 Additional health economic findings

8.1 Relative threshold unit intervention cost

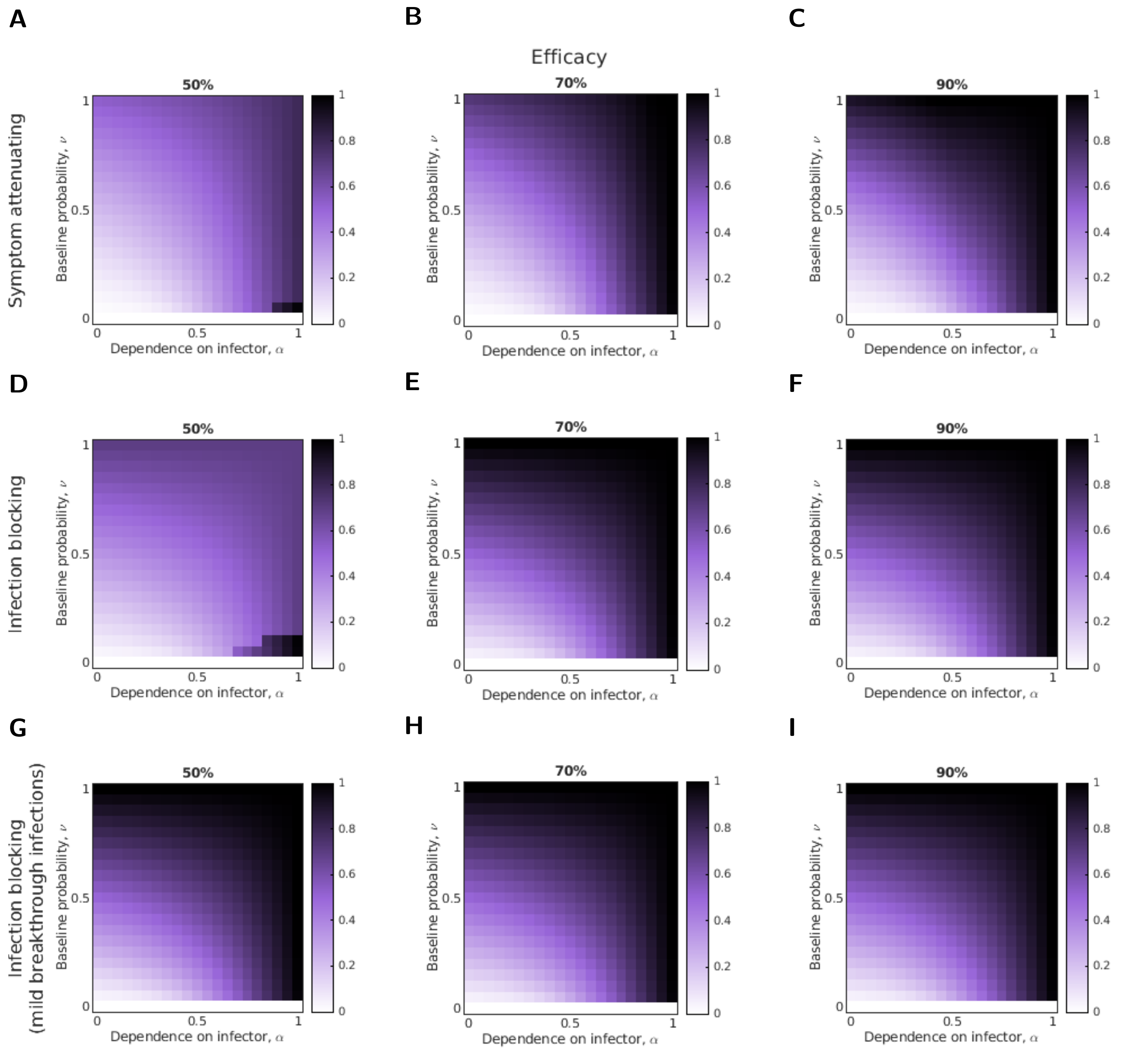

Figure X: Variation in the threshold unit intervention cost with α, ν and the efficacy for three intervention types, seasonal influenza-like parameters and a fixed intervention uptake (50%). Cell shading denotes the relative threshold unit intervention cost (normalised by the highest threshold unit intervention cost value). Darker shading corresponds to larger values. The assessed interventions had the following action: **(a-c)** symptom attenuating; **(d-f)** infection blocking; **(g-i)** infection blocking that only admits mild breakthrough infections. We performed evaluations for three efficacies per intervention: **(a,d,g)** 50%; **(b,e,h)** 70%; **(c,f,i)** 90%.

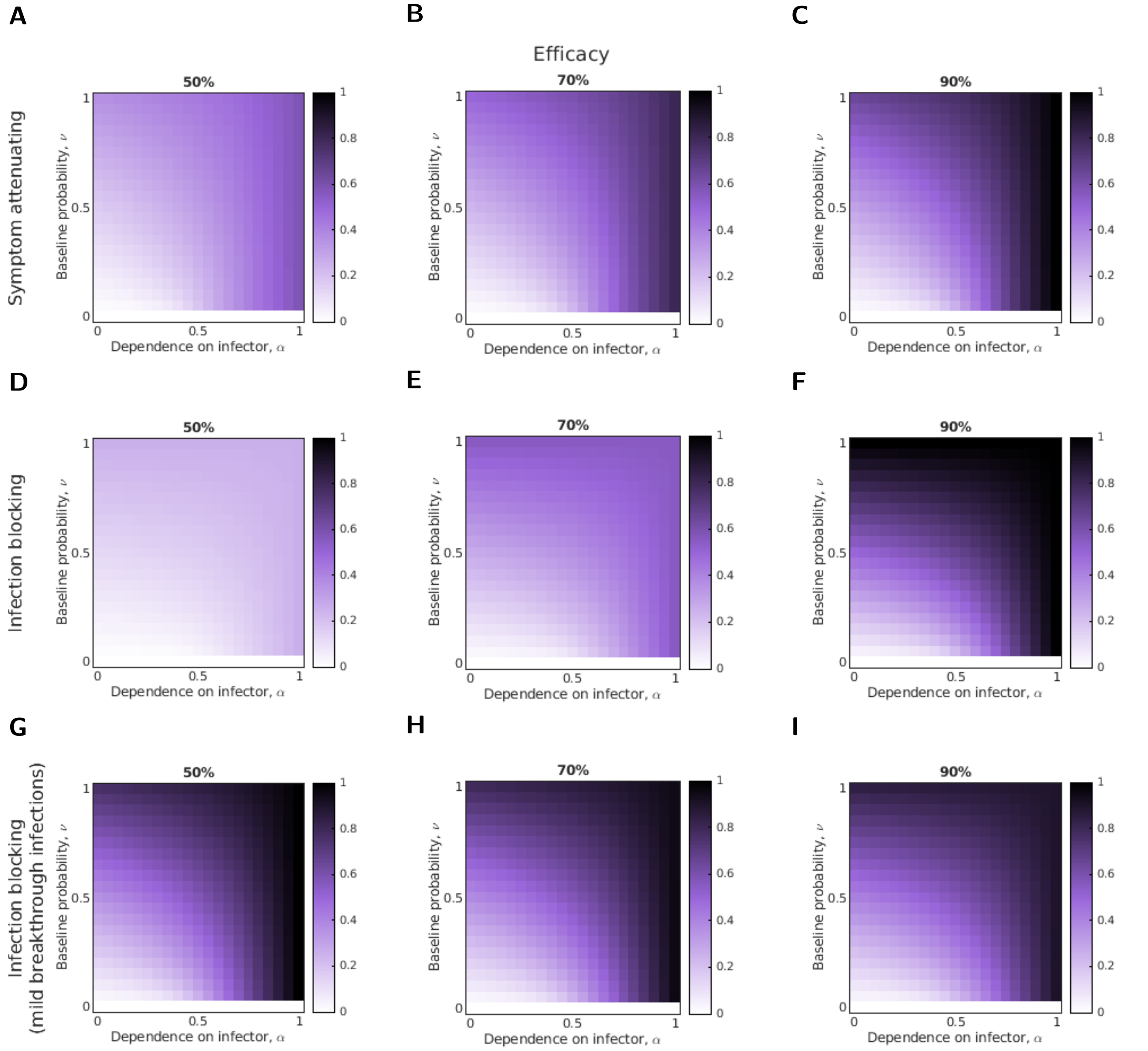

Figure Y: Variation in the threshold unit intervention cost with α, ν and the efficacy for three intervention types, pandemic influenza-like parameters and a fixed uptake (50%). Cell shading denotes the relative threshold unit intervention cost (normalised by the highest threshold unit intervention cost value). Darker shading corresponds to larger values. The assessed interventions had the following action: **(a-c)** symptom attenuating; **(d-f)** infection blocking; **(g-i)** infection blocking intervention that only admits mild breakthrough infections. We performed evaluations for three efficacies per intervention: **(a,d,g)** 50%; **(b,e,h)** 70%; **(c,f,i)** 90%.

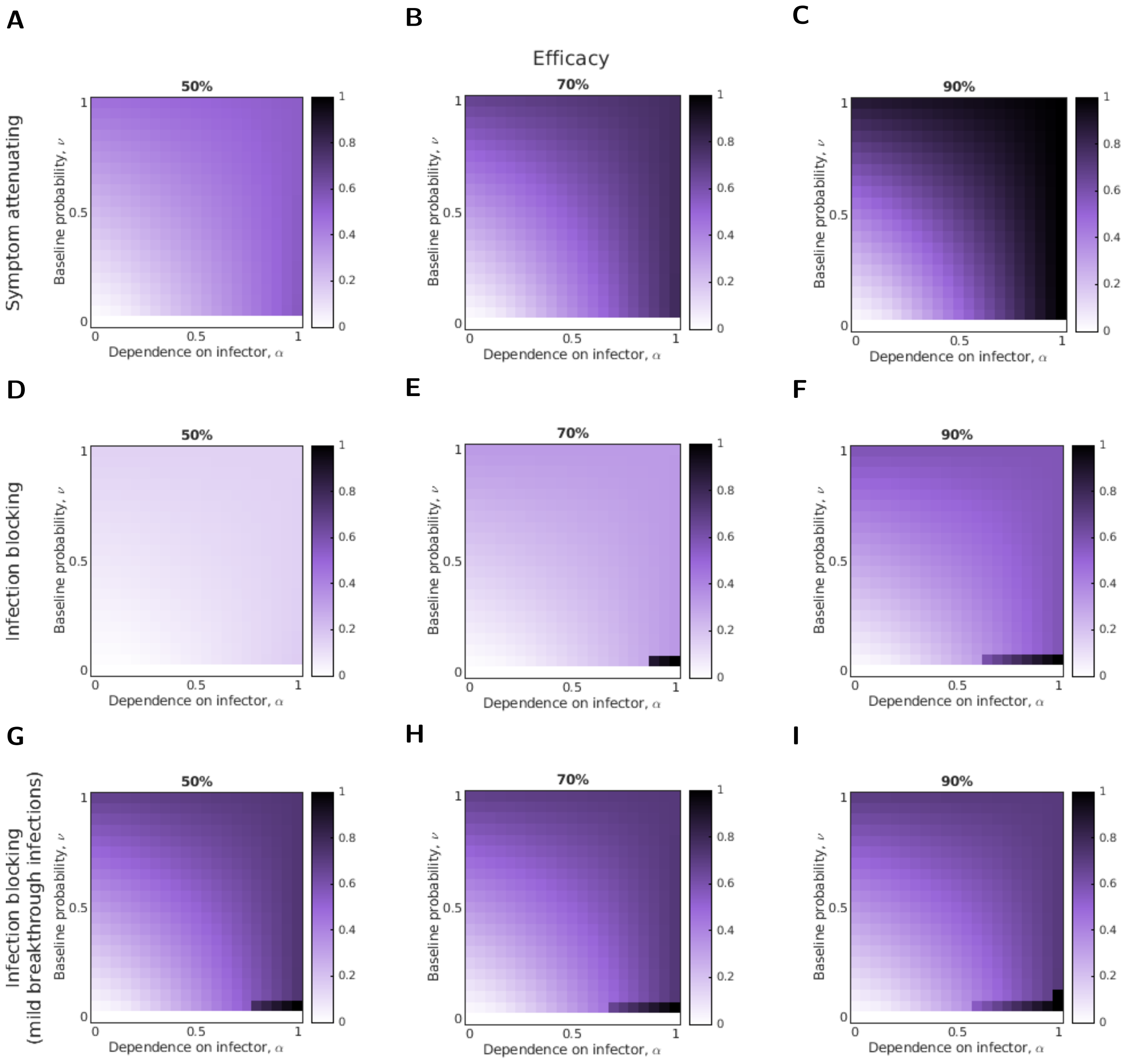

Figure Z: Variation in the threshold unit intervention cost with α, ν and the efficacy for three intervention types, SARS-CoV-2-like parameters and a fixed uptake (50%). Cell shading denotes the relative threshold unit intervention cost (normalised by the highest threshold unit intervention cost value). Darker shading corresponds to larger values. The assessed interventions had the following action: **(a-c)** symptom attenuating; **(d-f)** infection blocking; **(g-i)** infection blocking that only admits mild breakthrough infections. We performed evaluations for three efficacies per intervention: **(a,d,g)** 50%; **(b,e,h)** 70%; **(c,f,i)** 90%.

8.2 Most cost-effective intervention uptake

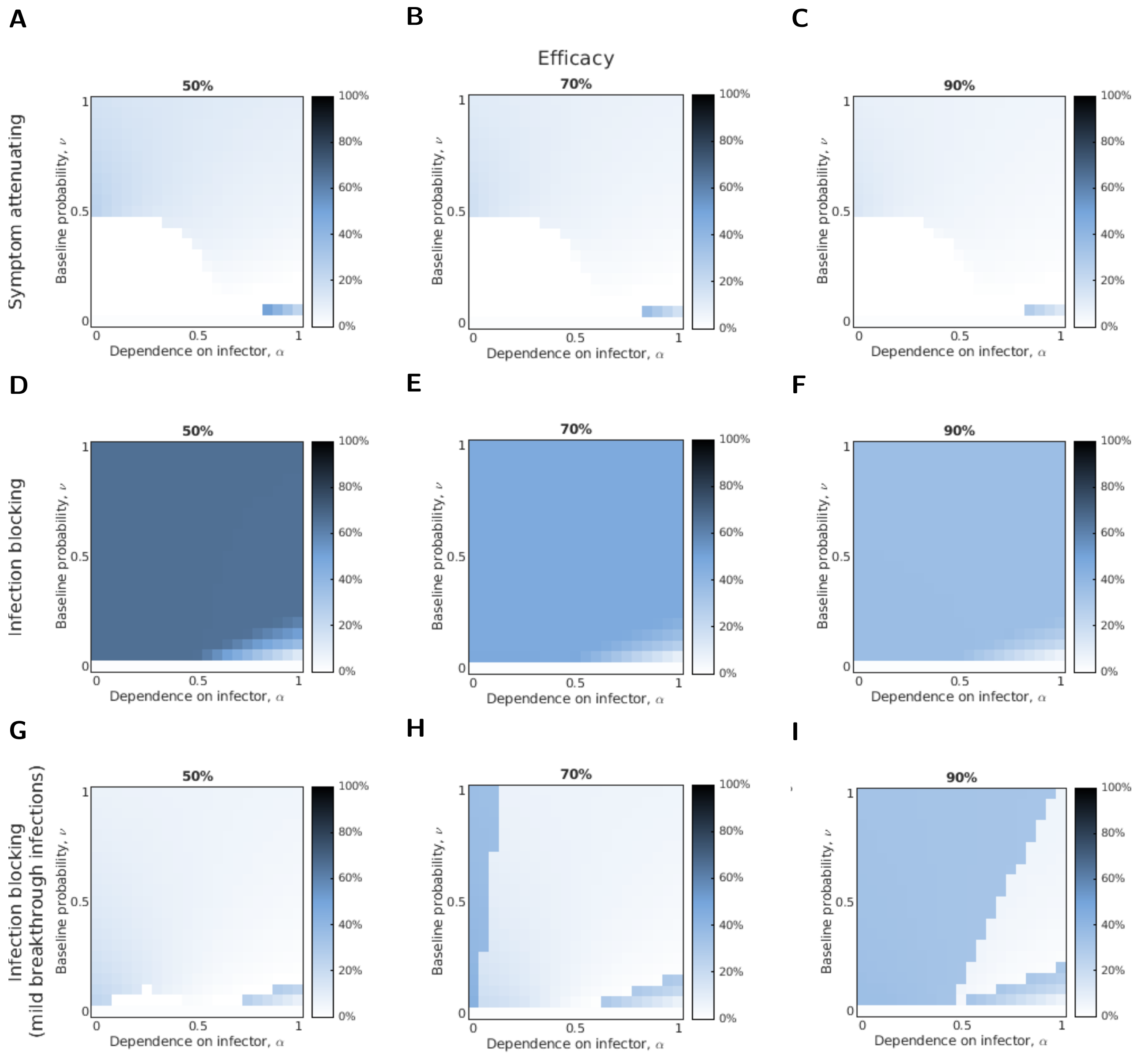

Figure AA: Variation in the most cost-effective vaccine uptake with α, ν and the efficacy for three intervention types and seasonal influenza-like parameters. Cell shading denotes the uptake at which the intervention was most cost-effective, i.e. the uptake at which the threshold unit intervention cost was at a maximum. Darker shading corresponds to larger values. The assessed interventions had the following action: **(a-c)** symptom attenuating; **(d-f)** infection blocking; **(g-i)** infection blocking that only admits mild breakthrough infections. We performed evaluations for three efficacies per intervention: **(a,d,g)** 50%; **(b,e,h)** 70%; **(c,f,i)** 90%.

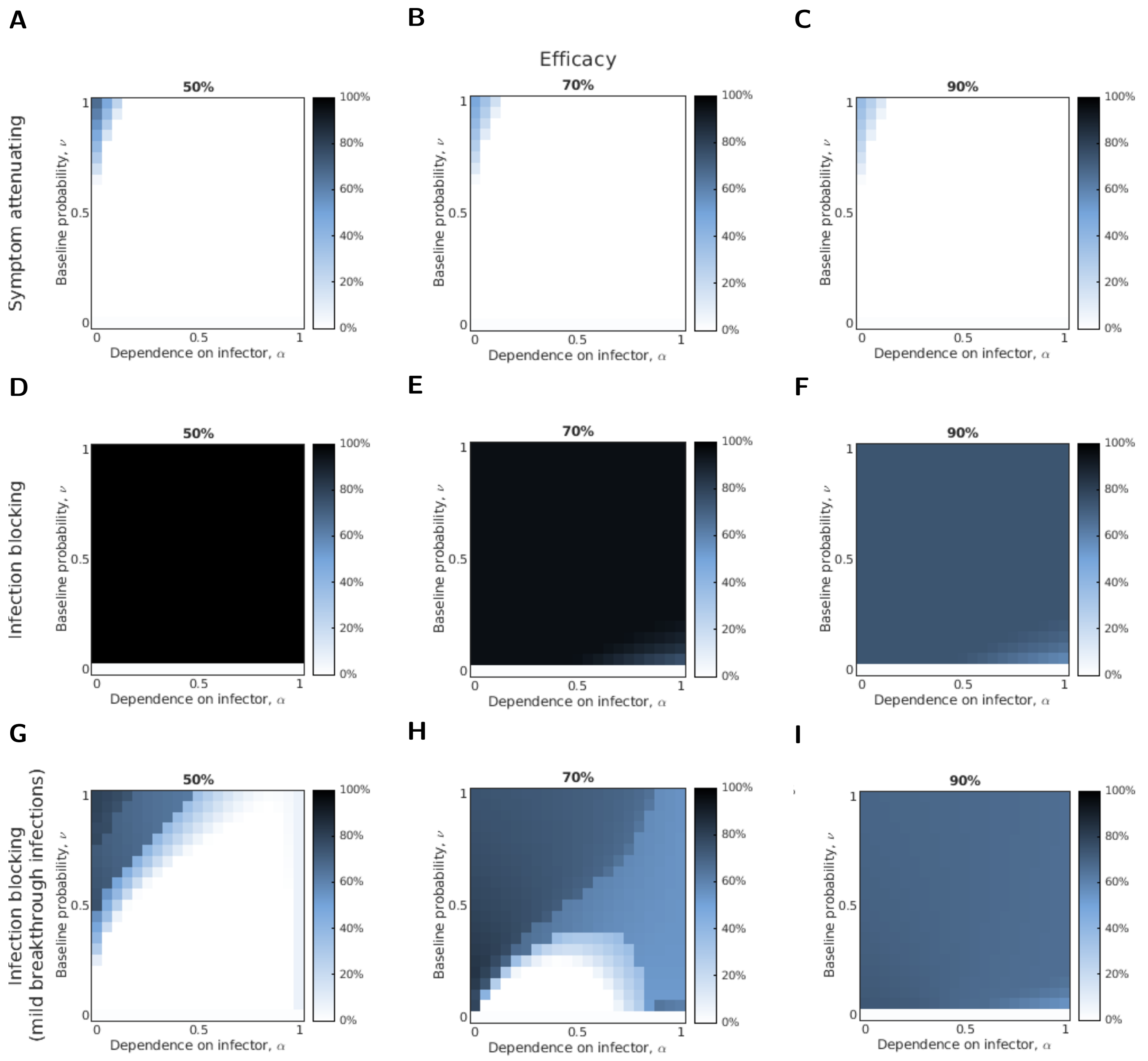

Figure AB: Variation in the most cost-effective vaccine uptake with α, ν and the efficacy for three intervention types and pandemic influenza-like parameters. Cell shading denotes the uptake at which the intervention was most cost-effective, i.e. the uptake at which the threshold unit intervention cost was at a maximum. Darker shading corresponds to larger values. The assessed interventions had the following action: **(a-c)** symptom attenuating; **(d-f)** infection blocking; **(g-i)** infection blocking that only admits mild breakthrough infections. We performed evaluations for three efficacies per intervention: **(a,d,g)** 50%; **(b,e,h)** 70%; **(c,f,i)** 90%.

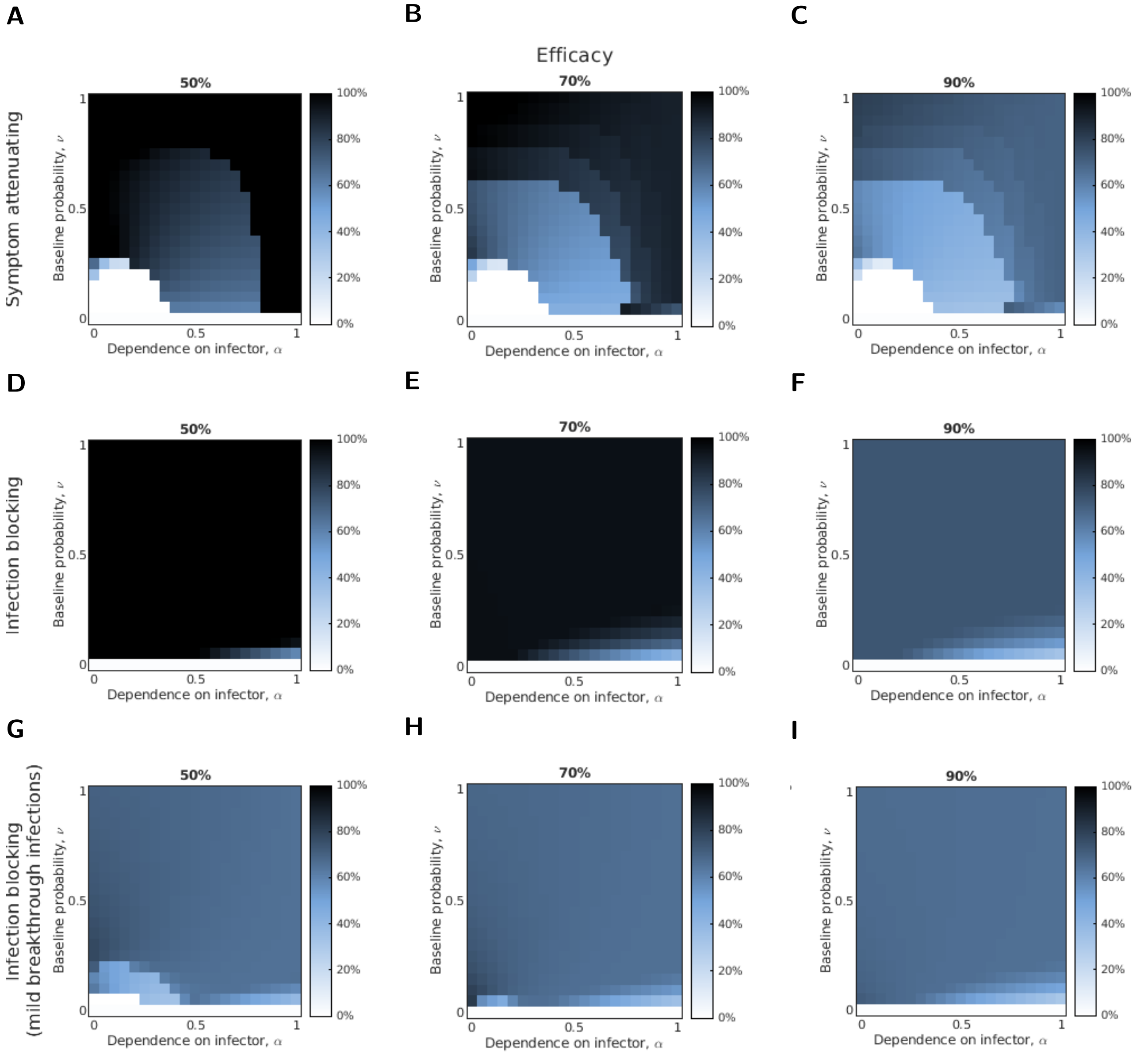

Figure AC: Variation in the most cost-effective vaccine uptake with α, ν and the efficacy for three intervention types and SARS-CoV-2-like parameters. Cell shading denotes the uptake at which the intervention was most cost-effective, i.e. the uptake at which the threshold unit intervention cost was at a maximum. Darker shading corresponds to larger values. The assessed interventions had the following action: **(a-c)** symptom attenuating; **(d-f)** infection blocking; **(g-i)** infection blocking that only admits mild breakthrough infections. We performed evaluations for three efficacies per intervention: **(a,d,g)** 50%; **(b,e,h)** 70%; **(c,f,i)** 90%.

8.3 Implication on threshold unit intervention cost when accounting for symptom propagation

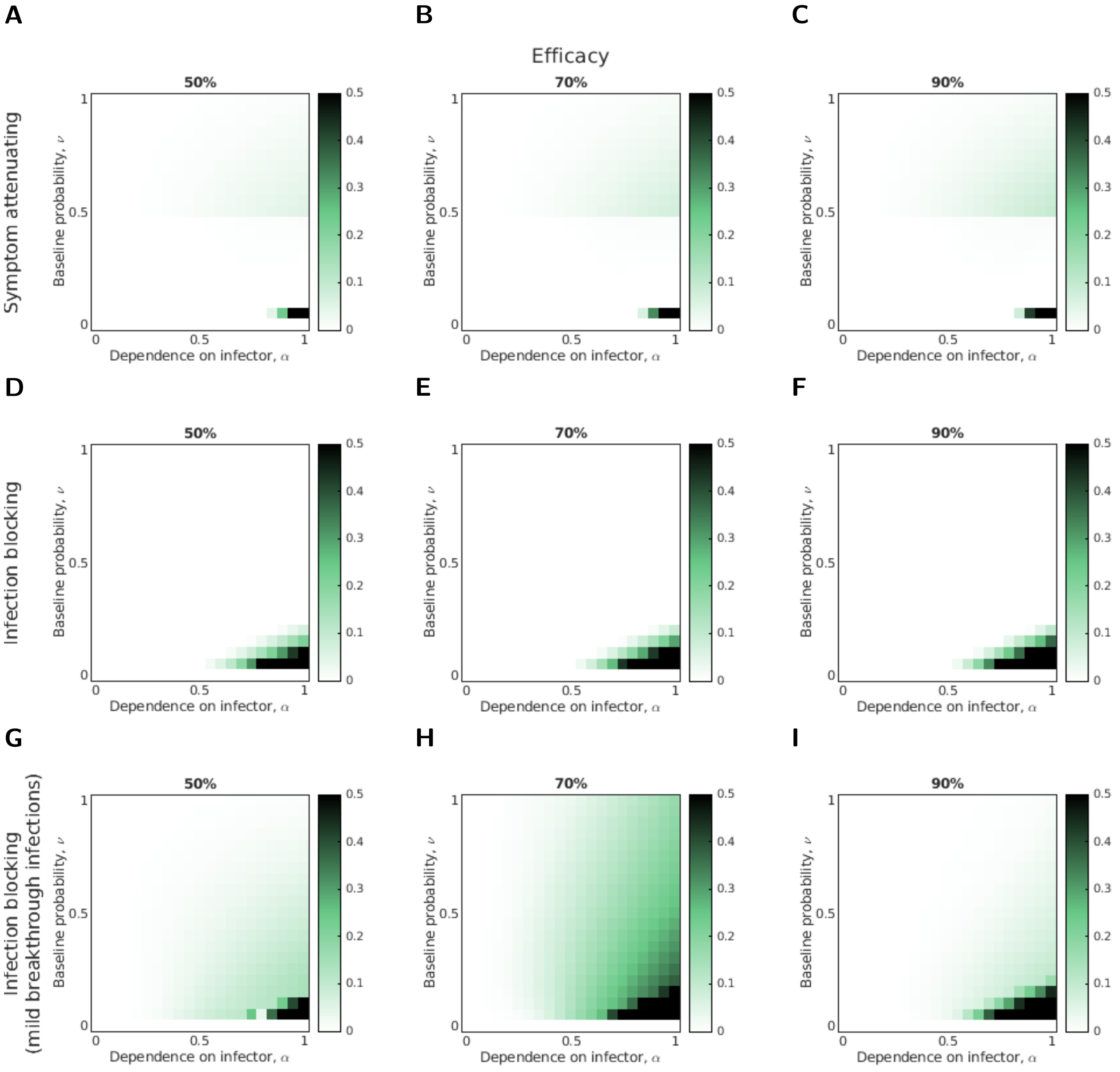

Figure AD: Comparing the threshold unit intervention costs at the most-effective uptake when accounting for symptom propagation to the threshold unit intervention cost at the most cost-effective uptake when there was no symptom propagation ($\alpha = 0$) for three intervention types and seasonal influenza-like parameters. In each panel, darker shading represents a greater disparity in the normalised threshold unit intervention costs between the most cost-effective uptake and the uptake that was most cost-effective when $\alpha = 0$. The assessed interventions had the following action: **(a-c)** symptom-attenuating; **(d-f)** infection-blocking; **(g-i)** infection-blocking that only admits mild breakthrough infections. We performed evaluations for three efficacies per intervention: **(a,d,g)** 50%; **(b,e,h)** 70%; **(c,f,i)** 90%.

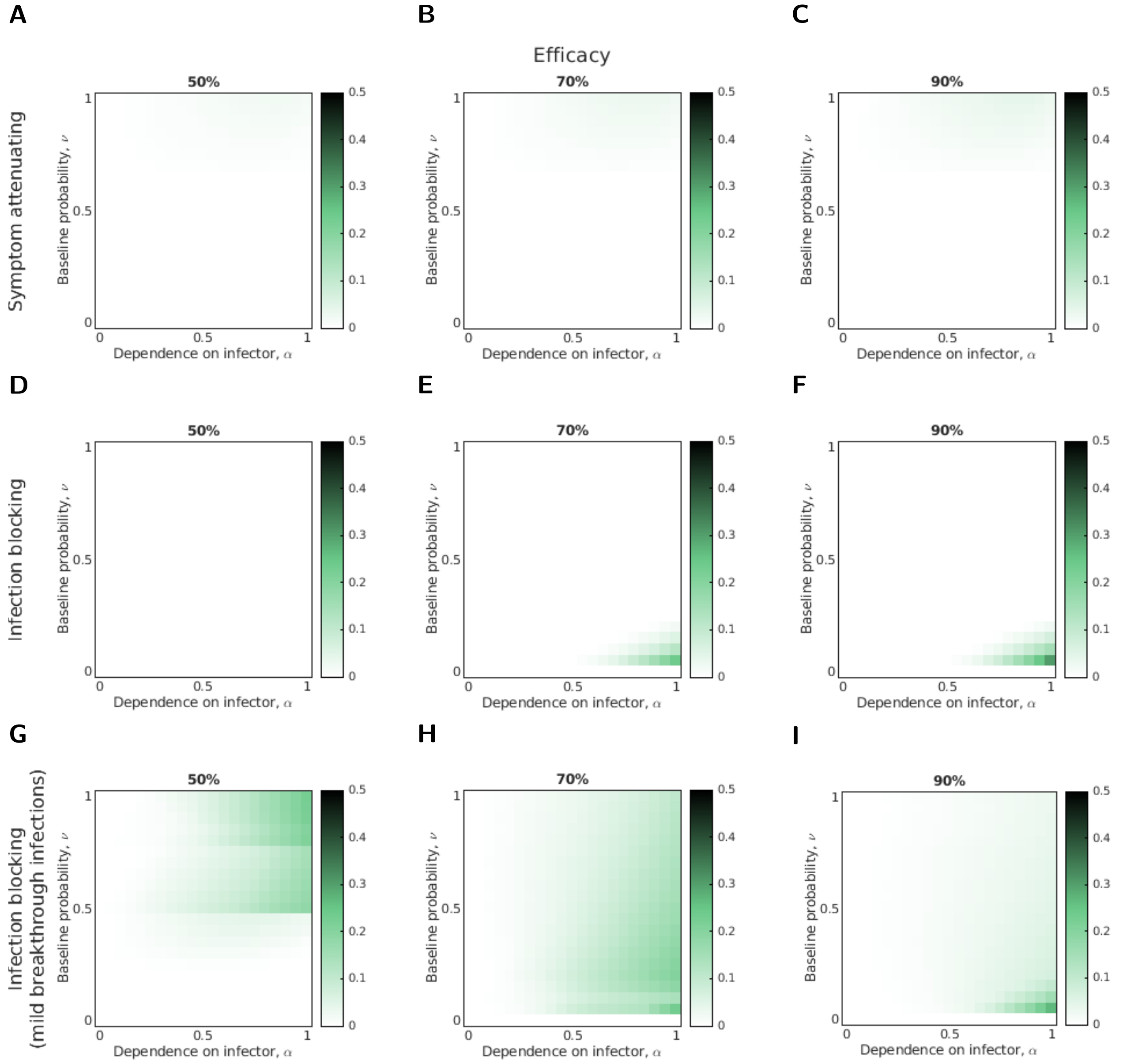

Figure AE: Comparing the threshold unit intervention costs at the most-effective uptake when accounting for symptom propagation to the threshold unit intervention cost at the most cost-effective uptake when there was no symptom propagation ($\alpha = 0$) for three intervention types and pandemic influenza-like parameters. In each panel, darker shading represents a greater disparity in the normalised threshold unit intervention costs between the most cost-effective uptake and the uptake that was most cost-effective when $\alpha = 0$. The assessed interventions had the following action: **(a-c)** symptom-attenuating; **(d-f)** infection-blocking; **(g-i)** infection-blocking that only admits mild breakthrough infections. We performed evaluations for three efficacies per intervention: **(a,d,g)** 50%; **(b,e,h)** 70%; **(c,f,i)** 90%.

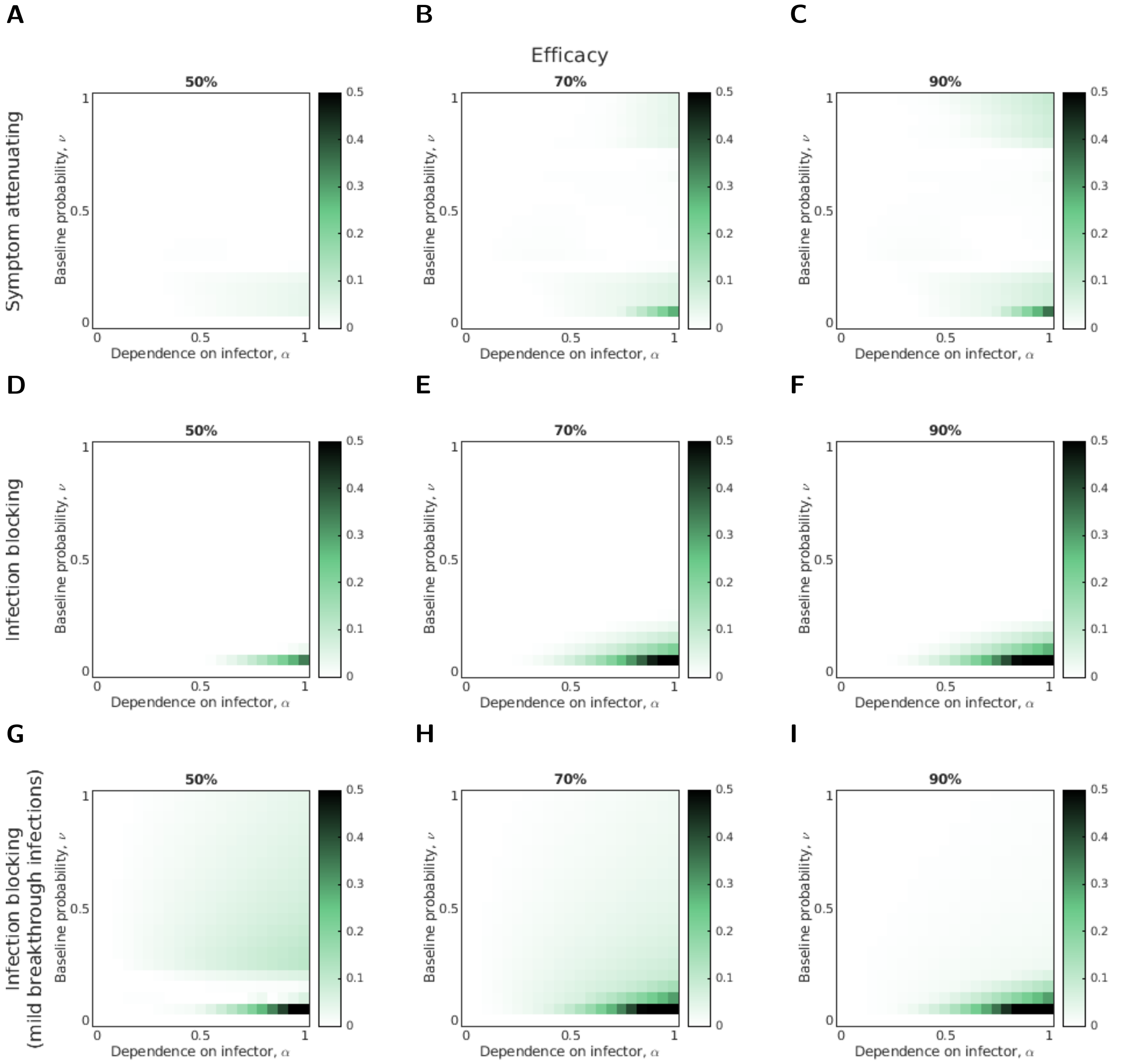

Figure AF: Comparing the threshold unit intervention costs at the most-effective uptake when accounting for symptom propagation to the threshold unit intervention cost at the most cost-effective uptake when there was no symptom propagation ($\alpha = 0$) for three intervention types and SARS-CoV-2-like parameters. In each panel, darker shading represents a greater disparity in the normalised threshold intervention costs between the most cost-effective uptake and the uptake that was most cost-effective when $\alpha = 0$. The assessed interventions had the following action: **(a-c)** symptom-attenuating; **(d-f)** infection-blocking; **(g-i)** infection-blocking that only admits mild breakthrough infections. We performed evaluations for three efficacies per intervention: **(a,d,g)** 50%; **(b,e,h)** 70%; **(c,f,i)** 90%.

References

[1] Diekmann O, Heesterbeek J, Metz J. On the definition and the computation of the basic reproduction ratio R 0 in models for infectious diseases in heterogeneous populations. *Journal of Mathematical Biology* **28**(4) (1990). doi:10.1007/BF00178324.

[2] Hill EM, Petrou S, Forster H, Lusignan Sd, Yonova I, *et al.* Optimising age coverage of seasonal influenza vaccination in England: A mathematical and health economic evaluation. *PLOS Computational Biology* **16**(10):e1008278 (2020). doi:10.1371/journal.pcbi.1008278. Publisher: Public Library of Science.

[3] Moran DP, Pires SM, Wyper GMA, Devleesschauwer B, Cuschieri S, *et al.* Estimating the Direct Disability-Adjusted Life Years Associated With SARS-CoV-2 (COVID-19) in the Republic of Ireland: The First Full Year. *International Journal of Public Health* **0** (2022). doi:10.3389/ijph.2022.1604699.

[4] Hollmann M, Garin O, Galante M, Ferrer M, Dominguez A, *et al.* Impact of Influenza on Health-Related Quality of Life among Confirmed (H1N1)2009 Patients. *PLoS ONE* **8**(3):e60477 (2013). doi:10.1371/journal.pone.0060477.

[5] Vekaria B, Overton C, Wiśniowski A, Ahmad S, Aparicio-Castro A, *et al.* Hospital length of stay for COVID-19 patients: Data-driven methods for forward planning. *BMC Infectious Diseases* **21**(1):700 (2021). doi:10.1186/s12879-021-06371-6.

[6] Lina B, Georges A, Burtseva E, Nunes MC, Andrew MK, *et al.* Complicated hospitalization due to influenza: results from the Global Hospital Influenza Network for the 2017–2018 season. *BMC Infectious Diseases* **20**(1):465 (2020). doi:10.1186/s12879-020-05167-4.

[7] Smith, DH, Gravelle. The practice of discounting in economic evaluations of healthcare interventions. *Int J Technol Assess Health Care* **17**(2):236–243 (2001). doi:10.1017/s0266462300105094.
